# Exploration of Specific Fluoroquinolone Interaction with SARS-CoV-2 Main Protease (Mpro) to Battle COVID-19: DFT, Molecular Docking, ADME and Cardiotoxicity Studies

**DOI:** 10.3390/molecules29194721

**Published:** 2024-10-05

**Authors:** Muhammad Asim Khan, Sadaf Mutahir, Muhammad Atif Tariq, Abdulrahman A. Almehizia

**Affiliations:** 1 School of Chemistry and Chemical Engineering, Linyi University, Linyi 276000, China; 2 Department of Chemistry, University of Sialkot, Sialkot 51300, Pakistan; 3 Department of Pharmaceutical Chemistry, College of Pharmacy, King Saud University, P.O. Box 2457, Riyadh 11451, Saudi Arabia

**Keywords:** fluoroquinolones, SARS-CoV-2 M^pro^, theoretical studies, DFT, molecular docking, ADME, cardiotoxicity

## Abstract

Herein, the pharmacokinetic profiles, binding interactions, and molecular properties of fluoroquinolone derivatives as prospective antiviral drugs are examined using a combination of docking, ADME, and DFT simulations. The effectiveness of the ligands is compared with the clinically tested and FDA-authorized medicine remdesivir. The findings demonstrated encouraging binding energies, indicating possible inhibitory effectiveness against SARS-CoV-2 M^pro^. The fluoroquinolone derivatives also exhibit promising ADME characteristics, although compounds 5, 6, 9, 12–20 possess poor values, suggesting that oral administration may be possible. The potential of the selected compounds as SARS-CoV-2 M^pro^ inhibitors is thoroughly understood because of the integrated analysis of DFT, with compound 11 demonstrating the highest energy gap of 0.2604 eV of, docking with viral targets with docking scores of −7.9 to −5.9 kcal/mol, with compound 18 demonstrating the highest docking score, which is at the 13th position in energy difference in the DFT data. Their favorable electrical properties, robust binding interactions with viral targets, and attractive pharmacokinetic profiles boost their potential as prospective study subjects. These substances have the potential to be transformed into cutting-edge antiviral therapies that specifically target SARS-CoV-2 M^pro^ and related coronaviruses.

## 1. Introduction

After SARS-CoV-2 broke out in December 2019, the WHO labeled it an infectious disease on 11 March 2020. This led to a global health emergency [1,2,3]. As of 11 June 2020, COVID-19 has spread all over the world; there have been more than 7 million reported cases and over 400 thousand confirmed deaths (World Meter, 11 June 2020). In reaction to the pandemic, numerous countries imposed strict lockdowns or nationwide isolations to slow the virus’s spread. As an outcome, more than one-third of the world’s population was subjected to various sorts of limitations (Business Insider, the seventeenth of April 2020) [4].

In the early studies conducted on COVID-19, it was discovered that the spike protein receptor-binding domain alongside the host receptor ACE2 was responsible for controlling the movement of the virus across species and its spread from one person to another, similar to what happened during the SARS-CoV-2 outbreak of 2020 [5,6]. At present, no particular vaccine or drug is suitable for COVID-19 treatment; however, numerous FDA-approved antimalarial and antiviral drugs have been anticipated, again as supportive care. As a result, the rapid creation of novel compounds for possible COVID-19 treatment has become a vital goal [7,8].

A recent study on SARS-CoV-2 has highlighted the importance of the M^pro^ hydrolase (chymotrypsin-like protease), referred to as the new coronavirus’s primary protease; 3-chymotrypsin-like protease (M^pro^ or 3CL^pro^) is the primary protease. This kind of protease is important in the coronavirus life cycle, and blocking the virus’s replication could be a promising treatment for COVID-19 [9]. Traditional Chinese medicine has gained popularity among Chinese people for the treatment of COVID-19. Because of their efficiency and cost-effectiveness, in silico methods such as molecular docking and molecular dynamics have become popular for identifying possible inhibitors for specific diseases [10]. Numerous scientists are conducting virtual screening with various potent libraries, including antiviral agents, antimalarial medications, plant sources, fungi, and synthetic substances, so that they can find suitable blockers for COVID-19 protease; one instance of this is a major protease (Mpro) of diseases like constant corona [11,12,13]. Computer-aided drug design, especially molecular docking and molecular dynamic simulations, has garnered much interest in drug development for specific disease treatments. For this purpose, we used the protein from the database with PDB-ID: 6LU7.

Fluoroquinolone antibiotics, based on their wide-ranging systemic effects, are useful in combating urinary tract and respiratory infections [14,15,16]. Gram-positive and Gram-negative aerobic bacteria, representing a wide range of species, can be affected by fluoroquinolones. These are used to alleviate infections caused by bacteria in humans, animals, and animal farming, notably chicken farming. Mostly all quinolone drugs are fluoroquinolones that contain a fluoro element in their chemical structure and are effective against bacteria [17,18,19]. Ciprofloxacin, one of the most extensively used medicines in the world, belongs to this class. Many fluoroquinolone analogs have been produced and licensed by the FDA in past years as wide-ranging defensive medicines used to cure respiratory and urinary bladder diseases [20,21].

Several widely viable fluoroquinolones are designed for treating infectious diseases and are effective in non-bacterial occurrences. Fluoroquinolones are also used to treat antiviral, antibacterial, and antimalarial-related diseases [22]. The derivatives of quinolones are also effective against biological action, such as displaying kinase, RAS, cardiotonic-sensitive PDE III inhibitory, anticancer, anti-inflammatory, antiallergic, antifungal, and antiparasitic effects [23]. Recently, ciprofloxacin and moxifloxacin have shown strong binding energy with SARS-CoV-2 M^pro^ [23,24,25]. As a conclusion, pulmonary fluoroquinolones could be utilized as an adjunct treatment in COVID-19 patients. The 20 fluoroquinolones were chosen based on the variety of their structural makeup and previous studies that suggested they would be effective antiviral drugs. Through the docking experiment, we hope to uncover new structural requirements for the effective binding and suppression of the viral protein [26,27,28].

**Figure 1:**
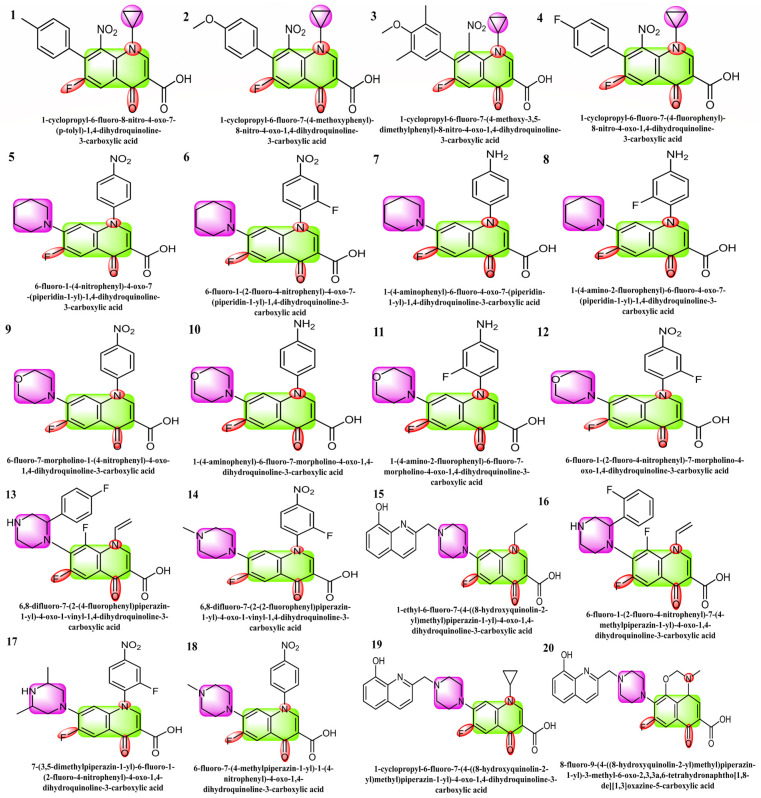
Structures **1** to **4** are the derivatives of fluoroquinolone with cyclopropyl substitutions, **5** to **8** are piperidine-substituted, **9** to **12** are morpholine-substituted, and **13** to **20** are piperazine-substituted fluoroquinolones.

The selected 20 fluoroquinolone-based compounds including carboxylic acid with cyclopropyl, piperidine-substituted fluoroquinolones, morpholine-containing fluoroquinolones, and piperazine-containing fluoroquinolones as shown in Figure 1, were investigated against SARS-CoV-2’s protease (M^pro^) [29,30,31]. The structural, electronic, in silico, ADME, and cardiotoxicity analyses/properties of these molecules have not been explored before by any research group. The present research mainly focused on the DFT studies of selected fluoroquinolone-based drugs and the binding of SARS-CoV-2 main protease with selected ligands and their toxicity studies. SARS-CoV-2 M^pro^ (PDB ID: 6LU7) was employed, which plays a vital role in viral replication [32,33,34]. The 96% sequence homology of SARS-CoV-2 M^pro^ is comparable to that of the prior SARS-CoV and resembles a dimer when complexed with N3, a peptide-like inhibitor [35,36]. Therefore, the selected 20 inhibitors were screened against SARS-CoV-2 M^pro^ by structure-based drug design.

Chemical reactivity, natural bond orbital analysis, equilibrium geometry, frontier molecular orbital analysis, and molecular electrostatic potential are some of the theoretical predictions that were investigated and computed using RCAM-B3LYP/6-311++G (2d, p) basis sets of DFT. The description of absorption, distribution, metabolism, and excretion (ADME), pharmacokinetics drug-likeness, and the cardiotoxicity of compounds were studied to explore the safety considerations for a new medicine, based on which risk-based evaluations can be made.

## 2. Results and Discussions

### 2.1. DFT Study

**Figure 2:**
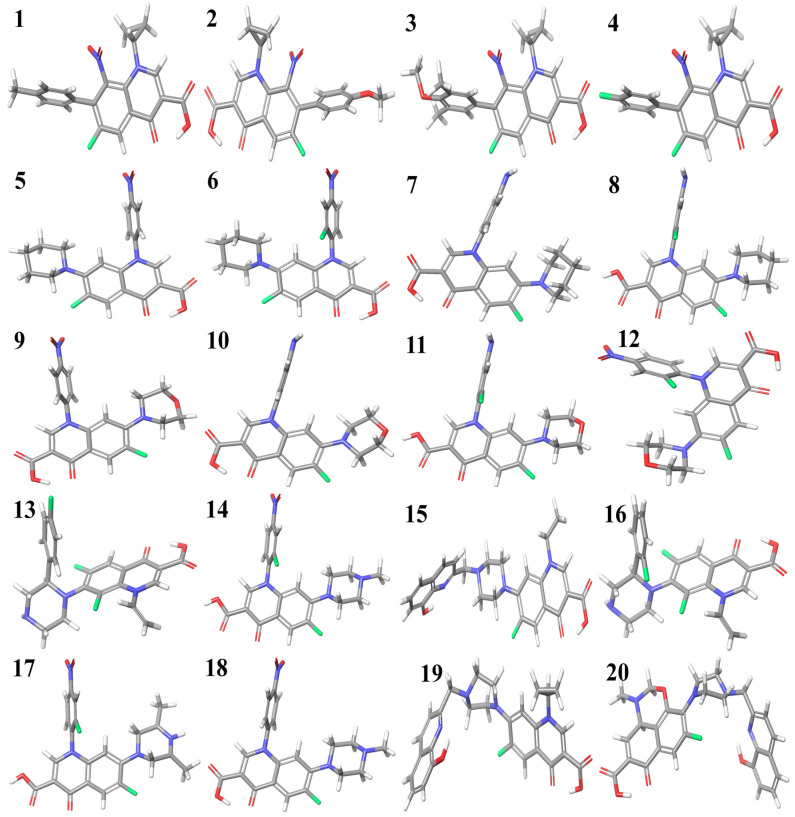
Optimized structures of fluoroquinolone derivatives with cyclopropyl (**1** to **4**), piperidine (**5** to **8**), morpholine (**9** to **12**), and piperazine substitutions (**13** to **20**).

The optimized structures of the selected compounds are shown in Figure 2. The geometries of the RCAM-B3LYP/6-311++G (2d, p) optimized clusters in the Cartesian coordinates of compounds **1**–**20** have been presented in the Supplementary Materials for researchers to reuse for future research purposes.

**Figure 3:**
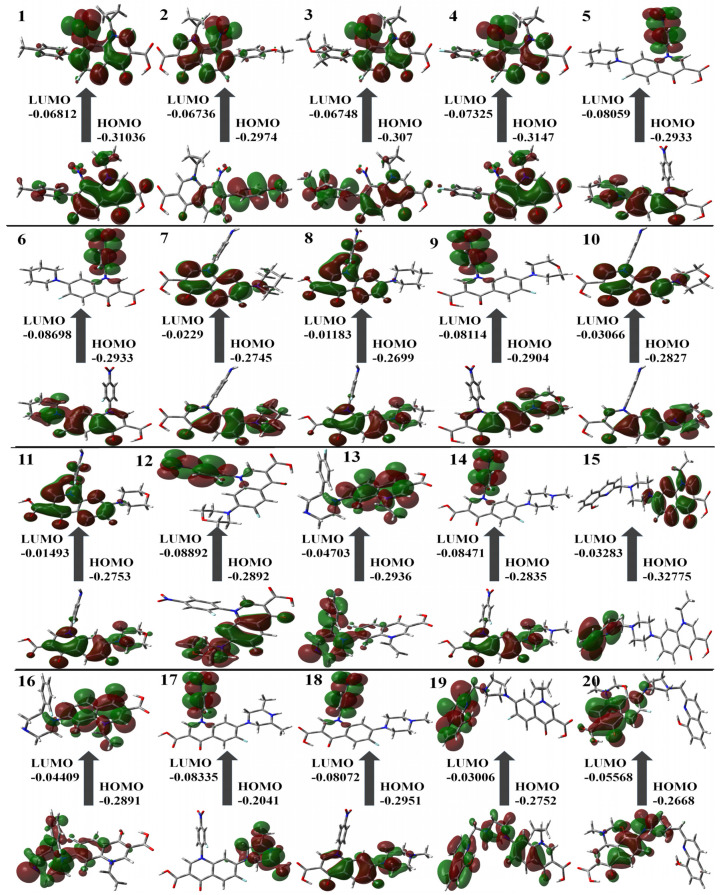
Molecular orbital surfaces and energy levels for the HOMO and LUMO of the compounds computed at RCAM-B3LYP/6-311++G (2d, p) basis set.

Table 1 contains the computed results for ionization potential, energy gap, electron affinity, and other descriptors. The stability of the structure is indicated by the energy gap; therefore, a ligand with a larger energy gap would be more stable compared to a molecule with a smaller gap. The order of the energy gaps for the selected compounds is **11** > **8** > **10** > **7** > **13** > **19** > **16** > **15** > **1** > **4** > **3** and so on. The largest energy gap of compound 11, according to the results, makes it more stable than the rest of the compounds due to the amine group shown in Figure 3. Compounds **4** and **11** possess the highest ionization potential, compound **12** shows the highest electron affinity, 11 possesses the highest hardness, **5**, **6**, **12**, and **20** are chemically softer as compared to others, 6 is highly electronegative, 16 has the highest chemical potential, 17 has highest electrophilicity index (all are in the mild range, N < 2) [37].

**Figure 4:**
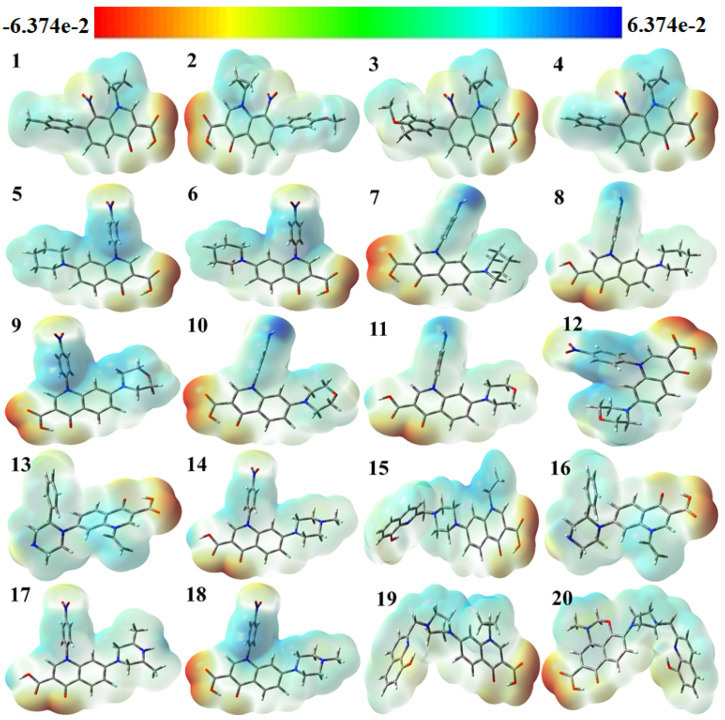
Molecular electrostatic potential (MEP) surfaces of selected compounds.

The use of color grading in MEP helps to indicate a molecule’s size, shape, and different possible charges, as shown in Figure 4 The colors are red, orange, yellow, green, and blue in descending order of potential. The blue color indicates the most electrostatically powerful area, meaning there are no electrons, while low electrostatic potential areas are indicated by the red hue, which has more electrons, hence making it an ideal place for an electrophilic attack [38]. With DFT calculations from the optimized structure and basis set as depicted in Figure 4, the surface analysis determined the MEP of the compounds. It is worth noting that gas-phase MEP surfaces have values ranging from −6.374 e^−2^ eV and +6.374 e^−2^ eV.

The hydroxyl and carbonyl groups of the selected fluoroquinolones have their oxygen atoms located in the most electrophilic area, as shown by the yellow region in Figure 4. From the MEP map, it can be inferred that the title molecules can work as antiviral molecules by entering a reaction system mainly through N atoms, because N atoms contain the nucleophilic part of the molecules, making them more suitable for reaction with negatively charged anions or radicals [39]. It was theoretically proved in 2005 by Hay et al. that, in the presence of an electron-withdrawing substituent, aryl C–H moieties become strong hydrogen bond donors and form stronger receptor–anion complexes than those of traditional hydrogen-bonding donor groups like N–H O–H, since nitro substitution can induce the receptors towards the binding of anions [40].

### 2.2. Molecular Docking Study

Molecular docking of selected compounds has been carried out through the MOE 2015 to find out their interaction with SARS-CoV-2 M^pro^, which is responsible for virus activity. Remdesivir has been used as a standard in this study. All except four ligands showed very efficient results compared to the standard and predicted results, indicating that these are very potent inhibitors of SARS-CoV-2 M^pro^. Their docking values demonstrated that these compounds can bind very efficiently with the main protease of SARS-CoV-2 M^pro^. Receptor–ligand interaction plays an important role in biological processes like the multiplication of microorganisms. The resultant data of the given compounds show that they can be used in the drugs and these are excellent inhibitors of SARS-CoV-2 M^pro^. Table 2 provides a summary of the outcomes from the effective docking of the investigated ligands (1–20).

### 2.3. Validation of MOE Software through Inhibitory Mechanism of N3

**Figure 5:**
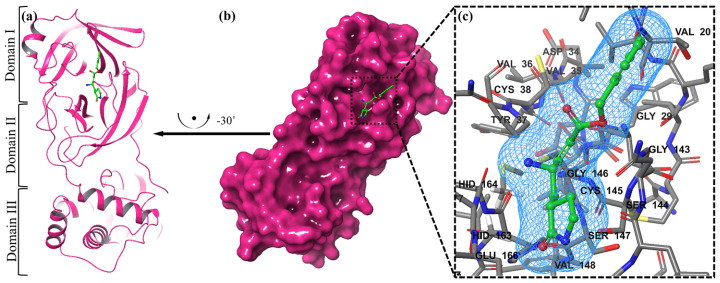
The representation of a single protomer from the SARS-inhibitor complex is depicted in a cartoon format (**a**). The homodimeric structure of SARS is shown in a surface representation. The protomer is shown by the pink colors, while the N3 inhibitor is depicted as green sticks (**b**). The substrate-binding pocket is highlighted in an enlarged view. Important binding pocket residues are represented as sticks. The density map (2Fo-Fc) contoured at 1.2σ is presented around the N3 molecule (blue mesh) (**c**).

We examined a crystal structure at 2.1 Å resolution that included SARS-CoV-2 Mpro together with an N3 compound, which is a Michael acceptor inhibitor developed using computer-aided drug design [7]. The crystal’s asymmetric unit contains a single polypeptide chain. Nonetheless, these polypeptide chains are designated as protomers through a crystallographic fold symmetry axis (Figure 5b). Notably, electron density maps confirm the visibility of all residues. The protomer is composed of three distinct domains (Figure 5a). The beta-barrel structures are formed by domains I (residues 1–123) and II (residues 123–194) in an antiparallel arrangement. The third domain (residues 195–306) has five alpha helices that usually appear to be arranged in an anti-parallel globular cluster, whereas its connection point to domain II is through a stretched loop region (residues 195–286). The catalytic dyad of Cys145-His41 can be found in SARS-CoV-2 Mpro, and the hydrolysis catalyzing center is located inside a gap between both domains I and II. These structural attributes bear a resemblance to previously reported SARS structures in other coronaviruses [7].

The electron density map clearly illustrates the binding of N3 within the substrate-binding pocket, adopting an extended conformation (Figure 5c). The inhibitor’s backbone atoms establish an antiparallel sheet formation, with the interconnecting loop between domain I on one side and domain II on the other.

Specific interactions between N3 and SARS-CoV-2 M^pro^ are elucidated in detail (Figure 5c). The electron density highlights the formation of a covalent bond between the Sγ atom of C145 in the protomer and the Cβ atom of the vinyl group, thus confirming the occurrence of Michael addition. This establishes a hydrogen bond with LEU 4 with ammonia in the protomer.

Previous studies have suggested a conserved substrate-recognition pocket within SARS-CoV-2 M^pro^, indicating its potential as a drug target for designing broad-spectrum inhibitors [7,41,42]. With the emergence of new types of coronaviruses and the accumulation of structural data for SARS-CoV-2 M^pro^ across diverse species, this hypothesis gains further support. Superimposing the crystal structures of SARS-CoV-2 M^pro^ with compound (N3) underscores the variability of helical domain I and surface loops while confirming the high conservation of the substrate-binding pocket located between domain I and domain II across all coronaviruses. This suggests that antiviral inhibitors targeting this pocket have the potential for broad-spectrum efficacy against coronaviruses (Figure 5) [43].

### 2.4. Visualization of Docking Results

**Figure 6:**
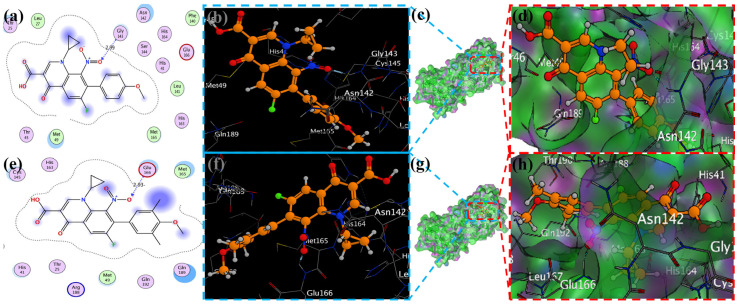
Docking with the 2 and 3 cyclopropyl ligands in the SARS-CoV-2 Mpro (6LU7). (**a**,**e**) The best binding mode of a protein (ligands 2 and 3, depicted as brown and red sticks), (**b**,**d**,**f**,**h**) The amino acid residues involved in bonding interactions (blue and green dashed line represents the ligand 2 and 3 interaction (**c**,**g**) (2D)).

**Figure 7:**
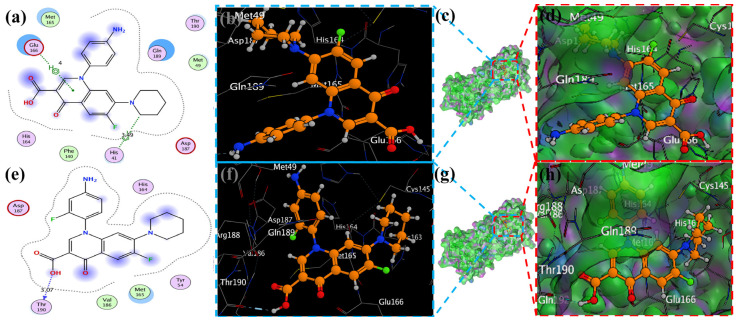
The piperidine ligands 7 and 8’s docking visualization in SARS-CoV-2 Mpro (6LU7); (**a**,**e**) show the optimal binding mode of the protein (ligands 7 and 8, depicted as brown and red sticks) while (**b**,**d**,**f**,**h**) highlight the amino acid residues engaged in bonding interaction (the blue and green dashed line represents the binding interaction (2D) of ligands 7 and 8 (**c**,**g**).

**Figure 8:**
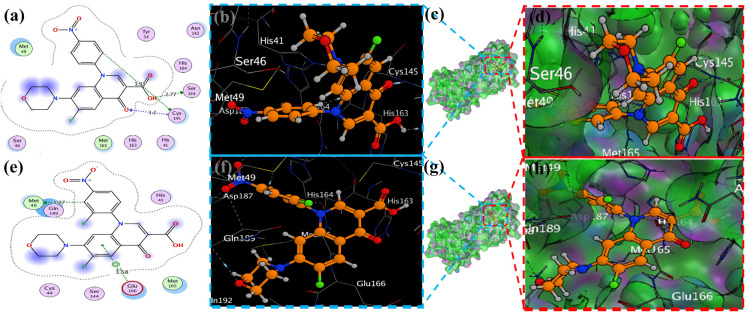
Docking representation of morpholine ligands 9 and 12 into SARS-CoV-2 Mpro (6LU7) (**a**,**e**): the most preferred binding configuration of the protein (ligands 9 and 12, shown as brown and red sticks, respectively) (**b**,**d**,**f**,**h**); interactions between amino acid residues participating in the bonding process (2D) ((**c**,**g**) show blue and green dashed lines, representing the binding interaction of ligands 9 and 12).

**Figure 9:**
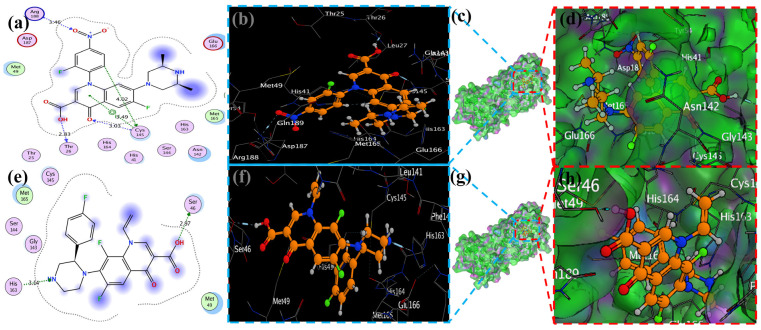
The docking of piperazine ligands 13 and 17 into SARS-CoV-2 Mpro (6LU7) was visualized as follows: (**a**,**e**) best binding mode of the protein (ligands 13 and 17, depicted as brown and red stick), (**b**,**d**,**f**,**h**). The amino acid residues involved in the bonding interaction (blue and green dashed lines represent the binding interaction (2D) of ligands 13 and 17 (**c**,**g**).

MOE has proven to be effective in creating 2D interactive ball-and-stick diagrams, as well as surface diagrams for selected compounds; a comparison was made with a standard FDA-approved drug (remdesivir). The docked result for the standard was −6.5, whereas these compounds demonstrated a potential docked score when compared with the standard. Compounds **2**, **3**, **8**, **11**, **14**, **15**, **19**, **20**, and **18** showed only one hydrogen bond at the Gly 143, Glu 166, Thr 190, Gly 143, Thr 190, Thr 25, Met 49, Thr 26, and His 163 residues with 2.99 Å, 2.93 Å, 3.07 Å, 3.04 Å, 2.84 Å, 3.8 Å, 4.23 Å, 3.22 Å and 3.48 Å in length, respectively (Figure 6, Figure 7 and Figures S1–S3). Compounds 13 and 9 formed two hydrogen bonds at Ser 46, His 163, Ser 144, and Cys 145 with a bond length of 2.97 Å, 3.14 Å, 2.77 Å, and 3.2 Å, respectively (Figure 8 and Figure 9).

Compound **17** formed three hydrogen bonds at the Thr 26, Cys 145, and Arg 188 residues with 2.83 Å, 3.03 Å, and 3.46 Å respectively (Figure 9). Compounds **1**, **4**, **9**, and **11** formed double bonds at the Glu 166, Glu 166, Cys 145, and His 41 residues with 4.42 Å, 4.3 Å, 3.93 Å and 4.44 Å, respectively (Figure 8, Figures S3 and S4). Similarly, compounds **14**, **17**, **7**, and **12** all showed two double bonds with Glu 166, Glu 166, Cys 145, Cys 145, His 41, Glu 166, Met 49, and Glu 166 residues, which were 4.39 Å, 3.73 Å, 3.49 Å, 4.02 Å, 3.49 Å, 4.0 Å, 3.27 Å and 3.58 Å in length, respectively (Figure 7, Figure 8 and Figure 9 and Figure S4). The remaining **5**, **6**, **16**, and **10** are compounds that could not show good results (Figures S5 and S6). When the compounds were docked with the protease (6LU7), it was discovered that the ligands fit perfectly in the core pocket region of the SARS-CoV-2 M^pro^ at the boundary between domain I and domain II, as shown in Figure 5 [42].

Table 3 presents the data of some of the best ligands against the docking results of FDA-approved and clinically tested antiviral and antimalarial drugs regarding the main protease of COVID-19 (6LU7). The binding energy data of remdesivir have been reported by researchers and are comparable with ligands 1, 3, 15, 17, and 18. The energy difference, ionization potential, electron affinity, chemical hardness, chemical softness, and electrophilicity index values are also comparatively better than other ligands, making them more suitable for binding with protein residues and also in protein pockets [44].

### 2.5. ADME Parameters, Pharmacokinetics, and Drug Likeness

The ADME (absorption, distribution, metabolism, and excretion) properties of twenty fluoroquinolone molecules as potential SARS-CoV inhibitors were analyzed in this study. The molecular weight (MW), hydrogen bond donors (HBD), hydrogen bond acceptors (HBA), QPlog Po/w, QPlogs, QPPCaco, metabolic stability, QPlog Khsa, and % age of human oral absorption were calculated using the QikProp command of the Schrodinger software, Maestro 11.2.

The results of the ADME analysis (Table 4) revealed significant variations among the selected fluoroquinolones. The molecular weight range should be from 130.0 to 725.0, and all selected molecules have values with the permitted range. Regarding the total number of hydrogen bonds donated by solute to water molecules in an aqueous solution (donor HB), the total number of hydrogen bonds accepted by solute from water molecules in an aqueous solution (accept HB), the predicted octanol/water partition coefficient (QP logPo/w), the predicted aqueous solubility (QP logS), the number of likely metabolic reactions (Metab), the prediction of binding to human serum albumin (Qplog Khsa), the predicted qualitative human oral absorption, and the predicted human oral absorption on a 0 to 100% scale, all values were within the permissible range, as mentioned in footnote of Table 4. In contrast, with the predicted apparent Caco-2 cell permeability in nm/sec, Caco-2 cells are a model for the gut–blood barrier; Caco-2 monolayers in the prediction of intestinal drug absorption range is <25 poor, >500 great, and the compounds **5**, **6**, **9**, **12**–**20**, showed poor intestinal absorption.

**Figure 10:**
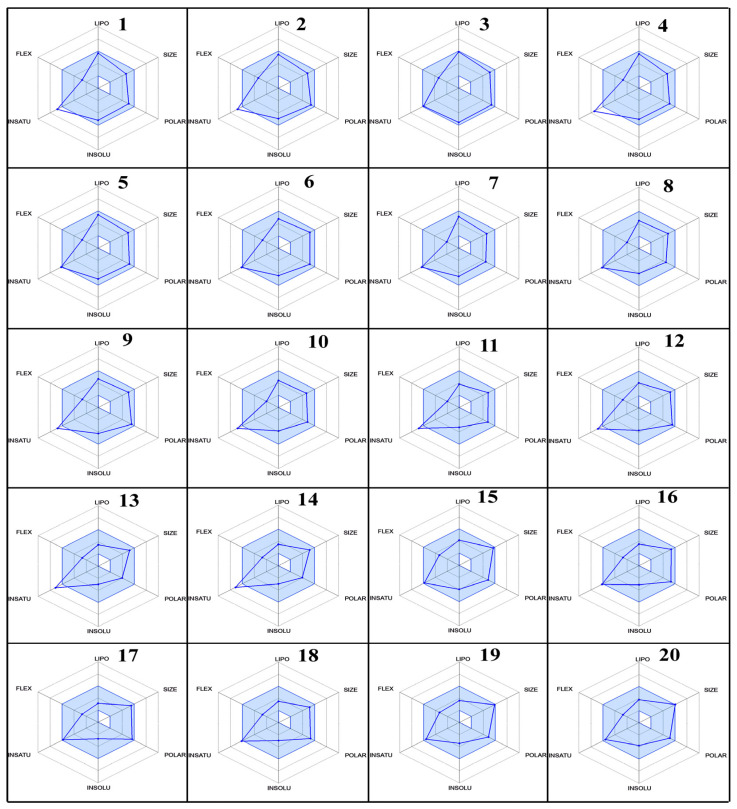
The radar plots of bioavailability data (lipophilicity, size, polarity, insolubility, instauration, and flexibility) of compounds **1**–**20**.

Furthermore, the % age of human oral absorption predictions indicated that all compounds had relatively high values, ranging from 25.64% to 79.41%. Compound **20** had the lowest % age of human oral absorption, while compound 3 showed the highest value. Lipinski’s rule of five showed that all compounds had a logP value less than 5, which is good for oral and intestinal absorption. These results provide valuable insights into the ADME properties of fluoroquinolones as SARS-CoV inhibitors with their radar (Figure 10), which was predicted by Swiss ADME online services. The variations in molecular weight, lipophilicity, metabolic stability, blood–brain barrier penetration, and oral absorption can aid in the selection and optimization of lead compounds for further development as effective antiviral agents.

### 2.6. Cardiotoxicity of Compounds

Pred-hERG 5.0 may provide information regarding the interaction of various compounds with hERG (the gene for human ether-a-go-go-related cardiac potassium channel), which is crucial in controlling heart rhythm fluctuations. The majority of compounds are classified as “non-blockers,” implying an expectation of minimal impact on the hERG channel. Confidence percentages accompanying these predictions vary from 67.29% to 99.83%, signifying diverse levels of certainty. The concept of “applicability domain” is introduced, with most compounds falling “Outside” this domain, suggesting potential limitations in prediction reliability. Categorical assessments of “Categorical Potency” further characterize the compounds, primarily as “Moderate blockers”, despite the overarching non-blocking prediction. Numerical “Potency” values add a quantitative dimension, showcasing the strength of potential interactions. Notably, the dataset highlights the model’s reliance on categorical assessments, with a tendency to predict non-blocking behavior even when categorical potency suggests otherwise. These findings underscore the need for the careful consideration of confidence levels, applicability domain, and categorical potency when interpreting predictions from Pred-hERG 5.0, enriching our understanding of the nuanced landscape of hERG channel interactions shown in Table 5.

**Figure 11:**
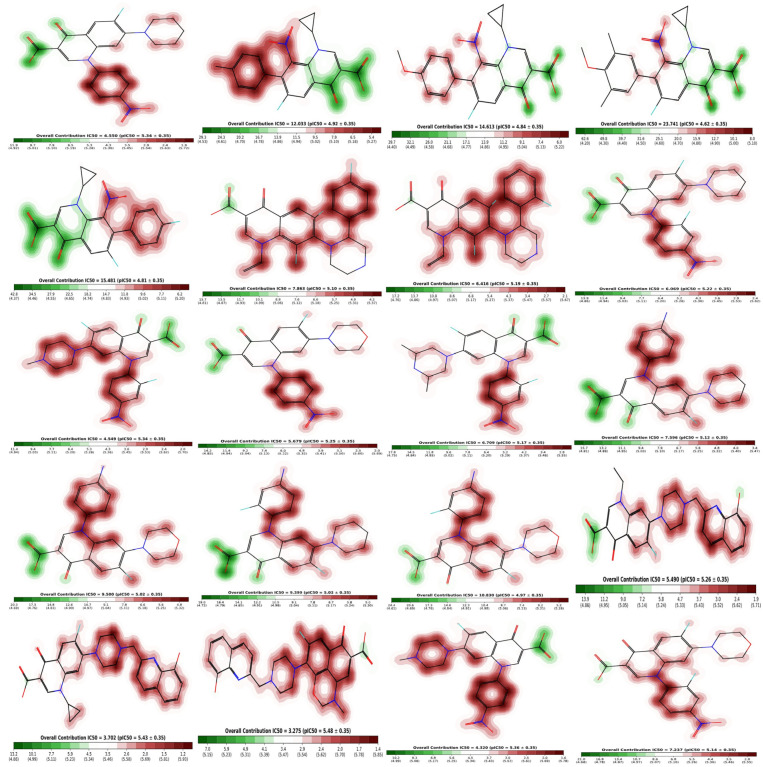
Fragment contribution maps for the regression model with IC_50_ and PIC_50_ values of compounds **1**–**20**.

The fragment contribution map produced by Pred-hERG for the most and least strong compounds is shown in Figure 11. Each atom is given a specific color based on how much it contributes to the activity. The colors red, red-orange, and orange are associated with the negative gradient contribution, whereas the colors yellow, yellow-green, and green are associated with the positive atomic contribution. In this study, we used an in silico technique to predict the cardiotoxicity of all substances. All compounds showed potential non-cardiotoxicity, except compounds **6** and **18**, which are potentially cardiotoxic and they are predicted as blockers.

## 3. Materials and Methods

The energy gap of analogs of fluoroquinolones was demonstrated by considering their HOMO-LUMO energies and the role they play in chemical interactions. The work also utilized molecular electrostatic potential to predict sites of nucleophilic and electrophilic attack on molecules, while several computational tools were applied to investigate their ability to inhibit SARS-CoV-2. The DFT calculations were performed using Gaussian 09 W Revision D.01, while binding affinity calculations were performed using MOE 2015, Pred-hERG was used for cardiotoxicity prediction, and Maestro 13.5 was utilized for calculating drug properties. This study explains the mechanism of action for the selected fluoroquinolones and their respective potentials of being therapeutic agents against SARS-CoV-2 via understanding their activities, ADME profiles, and cardio-toxicity risks [38,51,52,53,54,55].

### 3.1. Density Functional Theory (DFT) Study

We used DFT to determine the chemical hardness, ionization potential and softness, electrophilicity, and total energy of a process. The terms LUMO and HOMO refer to the lowest and highest occupied molecular orbitals, respectively. Molecules’ ionization energy and electron affinity are correlated with their HOMO and LUMO energies (Table 1) [56].

Koopmans’ theorem equation: A = −E_LUMO_ and I = −E_HOMO_

The energy difference reveals details about the molecules’ reactivity. While a lower energy difference suggests that a molecule is the least stable and most reactive, a bigger gap suggests that a molecule is extremely steady and less reactive. The electronic chemical potential is the name given to a structure’s negative electronegativity and is represented by the symbol µ.

µ = (E_HOMO_ − E_LUMO_)/2

This quantifies an electron’s ability to leave a system. A molecule is said to be more reactive and unstable when its value is greater. The ability of a system to draw an electron is gauged by the electrophilicity index [57]. This formula is used to compute it:

Ꞷ = µ^2^/2η

The solidity and reactivity of a compound are connected to chemical hardness. It calculates the degree of resistance to changes in the electron distribution [58].

η = (I − A)/2

The electronegativity **χ** of a molecule indicates its capacity to attract electrons.

χ = (IP + EA)/2

Tetracyanoethylene, the most electrophilic neutral molecule, has an index called nucleophilicity N that is correlated with its HOMO energy (TCE).

N = E_HOMO_ (nucleophile) − E_HOMO_(TCE)

With Gaussian 09 W Revision D.01, all the structures of compounds 1–20 have their energy minimized by DFT using the RCAMB3LYP functional and 6-31++G(2d, p) basis set in the DMSO solvent. A similar approach has been used for Gauss view version 5 for MEP computations. Using the principles of quantum mechanics, Gaussian makes predictions about molecular structure and spectroscopic data. The Gauss view and Gaussian09 were used to calculate the different energies of the selected substances. The Hartree–Fock approach, simulation methods, molecular mechanics, and post-Hartree–Fock investigations utilizing density functional theory (DFT) are all used to investigate various structural and electrical properties of organic molecules [32,33]. The well-known HOMO-LUMO energies of quantum mechanics influence numerous chemical interactions. One of the more beneficial discoveries for explaining a molecule’s chemical stability is the FMOs theory (Frontier molecular orbitals), which uses HOMO and LUMO. The MEP of the selected structures has been predicted using the Gauss view software 09 [34,59,60].

### 3.2. Docking Study Using MOE (Molecular Operating Environment)

Initially, the MPro: N3 complex’s crystal structure (PDB ID: 6LU7; Version 2, 2.16 Å resolution, 4) was downloaded. Although Mpro typically operates as a homodimer, as mentioned in the Introduction, we only computed the monomeric unit found in the PDB file, because the dimer interface does not directly interact with the N3 ligand. Using the MOE tool, we further processed the molecular structure. The 84 crystal water molecules were all saved, and the FMO calculation was then performed on them. The peptide-like N3 inhibitor is shown in Figure 5a as the protein’s ligand. Take note that the Michael addition has given this ligand a covalent connection with Cys145. Standard modeling activities, such as pKa adaption (pH 7) and hydrogen attachment, were carried out. Ser1 and Gln306 were given the charged N- and C-termini, respectively. Within all attached hydrogen atoms, the ligand, and the amino acid residues within 4.5 Å of the ligand, molecular mechanics-based energy minimization (using the AMBER10:EHT force field in MOE) was performed; a tether mask of 1.0 Å was employed within the important pharmacophore region. Figure 5b displays the whole structure of the processed protein–ligand complex along with a close-up of the pharmacophore. Based on the geometrical relationships determined by MOE, Figure 5c is a schematic representation of the interactions between ligands and residues. [61,62].

### 3.3. ADME Studies

The Maestro Schrodinger and Swiss ADME online services have been used to forecast the substances’ assessment of absorption, distribution, metabolism, and excretion (ADME). Using a large database, the server may make high-precision assumptions about physicochemical attributes, pharmacokinetics, drug similarity, and different pharmacological effects. For the ADME study, the selected compounds, after optimization by DFT, were used in the Maestro Schrodinger and Swiss ADME services, which gives the ADME values in the form of table and radar form [63].

### 3.4. Cardiotoxicity Studies of Compounds

Pred-hERG, a web-enabled computer framework, was utilized to calculate the prediction, confidence, and probability map for all the drugs’ potential cardiac toxicity. The Pred-hERG server aids users in identifying hERG blockers and non-blockers by providing a quick, user-friendly interface. We used an optimized compound from DFT in CHK file for cardiotoxicity [64].

## 4. Conclusions

In conclusion, this study used computational chemistry methods to assess the potential of several fluoroquinolone compounds as SARS-CoV-2 M^pro^ inhibitors. The molecular characteristics of the chosen compounds, such as their HOMO and LUMO energies, ionization potential, energy gap, electron affinity, and other descriptors were well understood by DFT analysis. The more stable properties of the compounds with bigger energy gaps point to their potential as inhibitors. The selected compounds’ electron distribution and reactivity were further shown by the HOMO and LUMO plots. An anti-aromatic molecule was suggested by a tiny HOMO-LUMO gap, whereas a hard, stable, and less reactive molecule was suggested by a broad gap. These results help us understand the stability and chemical behavior of fluoroquinolone derivatives. The molecular electrostatic potential (MEP) analysis also made it possible to locate the molecules’ nucleophilic and electrophilic attack sites. The visual representation of the positive, negative, and neutral regions offered by the MEP surfaces allowed for the evaluation of possible interactions and inhibitory capacities. Furthermore, the molecular docking analyses of all compounds except six, utilizing MOE, showed the chosen compounds’ propensities for attaching to the SARS-CoV-2 M^pro^ with the docking score between −7.9 kcal/mol and −5.9 kcal/mol. The effectiveness of the compounds in attaching to the main protease of SARS-CoV-2 M^pro^ was shown by the docking scores. Numerous substances outperformed remdesivir in terms of docking scores, indicating that they could be effective SARS-CoV-2 M^pro^ inhibitors. The chosen fluoroquinolone compounds appear to have potential as SARS-CoV-2 M^pro^ inhibitors, according to the combined findings of the DFT analysis, HOMO-LUMO plots, MEP analysis, and molecular docking investigations. These results offer useful information for future research and the creation of possible antiviral medications to treat COVID-19 infections. The obtained result of the ADME study showed the pharmacokinetics and drug-likeness of these compounds, where all the ligands showed good physicochemical properties, lipophilicity, water solubility, pharmacokinetics, drug-likeness, and medicinal chemistry features. The ligands 1, 3, 15, 17, and 18 showed better electronic, structural, in silico, ADME and cardiotoxicity properties, so these fluoroquinolone compounds have a good scope for further drug development.

## Figures and Tables

**Table 1:** The basis set, RCAM-B3LYP/6-311++G (2d, p), was used to compute the DFT level descriptors for selected compounds for the HOMO-LUMO energy differences.

Comp	*E*_LUMO_ (eV)	*E*_HOMO_ (eV)	∆E _(HOMO-LUMO)_ (eV)	Ionization Potential (*I*) (eV)	Electron Affinity (*A*) (eV)	Chemical Hardness (*η*) (eV)	Chemical Softness (*ζ*) (eV)	ELECTRONEGATIVITY (*χ*) (eV)	Chemical Potential (*μ*) (eV)	Electrophilicity Index (*ω*) (eV)
**1**	−0.3103	−0.0681	0.2422	0.3104	0.0681	0.1211	4.1281	0.1892	−0.3440	0.0072
**2**	−0.2973	−0.0673	0.2300	0.2974	0.0674	0.1150	4.3474	0.1823	−0.3310	0.0063
**3**	−0.3069	−0.0674	0.2395	0.3070	0.0675	0.1197	4.1755	0.1872	−0.3400	0.0070
**4**	−0.3147	−0.0732	0.2415	0.3147	0.0733	0.1207	4.1411	0.1939	−0.3510	0.0075
**5**	−0.2933	−0.0805	0.2127	0.2933	0.0806	0.1064	4.7006	0.1869	−0.3330	0.0059
**6**	−0.2932	−0.0869	0.2063	0.2933	0.0870	0.1031	4.8475	0.1901	−0.3360	0.0058
**7**	−0.2745	−0.0229	0.2516	0.2745	0.0229	0.1258	3.9739	0.1487	−0.2850	0.0051
**8**	−0.2698	−0.0118	0.2581	0.2699	0.0118	0.1290	3.8751	0.1408	−0.2750	0.0049
**9**	−0.2904	−0.0811	0.2093	0.2904	0.0811	0.1046	4.7785	0.1857	−0.3300	0.0057
**10**	−0.2826	−0.0306	0.2520	0.2827	0.0307	0.1260	3.9683	0.1566	−0.2970	0.0056
**11**	−0.2752	−0.0149	0.2604	0.2753	0.0149	0.1302	3.8408	0.1451	−0.2820	0.0052
**12**	−0.2892	−0.0889	0.2003	0.2892	0.0889	0.1002	4.9925	0.1890	−0.3330	0.0056
**13**	−0.2930	−0.0470	0.2466	0.2936	0.0470	0.1233	4.0556	0.1703	−0.3170	0.0062
**14**	−0.2834	−0.0847	0.1988	0.2835	0.0847	0.0994	5.0312	0.1840	−0.3250	0.0053
**15**	−0.2775	−0.0328	0.2447	0.2775	0.0328	0.1224	4.0865	0.1551	−0.2930	0.0053
**16**	−0.2891	−0.0440	0.2450	0.2891	0.0441	0.1225	4.0810	0.1666	−0.3110	0.0059
**17**	−0.2040	−0.0833	0.1207	0.2041	0.0834	0.0604	8.2850	0.1437	−0.2450	0.0018
**18**	−0.2950	−0.0807	0.2144	0.2951	0.0807	0.1072	4.6650	0.1879	−0.3350	0.0060
**19**	−0.2752	−0.0300	0.2451	0.2752	0.0301	0.1225	4.0793	0.1526	−0.2900	0.0052
**20**	−0.2668	−0.0556	0.2111	0.2668	0.0557	0.1056	4.7366	0.1612	−0.2940	0.0046

**Table 2:** Docking score (kcal/mol) of selected compounds with SARS-CoV-2 (6LU7).

Ligand	Docking Score kcal/mol	Predicted Inhibitory Constant (pK*_i_*) µM	∆G Energy kcal/mol
1	−6.4577	2.3159	−34.8948
2	−6.1160	0.9924	−32.3975
3	−7.4168	0.8129	−43.3306
4	−5.8692	0.8136	−31.0542
5	−7.5820	1.7860	−45.1559
6	−7.2341	3.1690	−40.8352
7	−6.8824	2.7863	−35.2465
8	−7.2390	1.4395	−37.6927
9	−7.2094	1.4301	−41.1037
10	−6.8317	2.1947	−41.7742
11	−6.9915	1.3738	−35.5844
12	−7.1314	1.1245	−34.9606
13	−6.5379	3.4226	−41.3863
14	−7.3066	2.6392	−41.6989
15	−7.4876	0.8797	−42.5053
16	−7.2495	2.4672	−41.5726
17	−7.5876	2.2114	−43.3508
18	−7.8704	1.9804	−48.5983
19	−7.2012	2.6694	−41.3393
20	−7.2246	2.3682	−41.6298

**Table 3:** A comparison of some of the best ligands with FDA-approved and clinically tested drugs to treat the main protease of COVID-19.

Compounds (Drugs)	Binding Energy, ∆G (kcal/mol)
Remdesivir	−5.8 [45], −7.215 [46], −2.47 [47], −6–5 [48], −9.70 [49], −7.5 [45], −3.62 [47], −5.1 [48], −5.75 [50].
Ligand 01	−6.4577 (this work)
Ligand 03	−7.4168 (this work)
Ligand 17	−7.3066 (this work)
Ligand 15	−7.4876 (this work)
Ligand 18	−7.8704 (this work)

**Table 4:** In silico absorption, distribution, metabolism, and excretion (ADME) study of manufactured molecules.

Title	Mol MW	Donor HB	Accept HB	QP logPo/w	QP logS	QPP Caco	Metab	Qplog Khsa	Human Oral Absorption	Percent Human Oral Absorption	Rule of Five	Rule of Three
1	382.347	0.00	4.50	3.511	−4.453	43.305	2	0.174	3	76.795	0	0
2	398.347	0.00	5.25	3.206	−3.906	43.379	2	−0.041	3	75.024	0	0
3	426.4	0.00	5.25	3.863	−4.918	46.489	4	0.278	3	79.405	0	0
4	386.311	0.00	4.50	3.436	−4.245	43.157	1	0.052	3	76.329	0	0
5	411.389	0.00	5.50	3.482	−5.576	12.778	1	0.314	2	67.138	0	1
6	429.379	0.00	5.50	3.647	−5.757	13.127	1	0.344	2	68.313	0	2
7	381.406	1.50	5.50	3.482	−5.586	26.62	1	0.381	2	72.839	0	0
8	399.396	1.50	5.50	3.654	−5.813	27.321	1	0.411	2	74.053	0	1
9	413.361	0.00	7.20	2.38	−4.211	12.778	2	−0.266	2	60.682	0	1
10	383.378	1.50	7.20	2.551	−4.609	26.62	2	−0.03	2	67.393	0	0
11	401.369	1.50	7.20	2.725	−4.84	27.321	2	−0.001	2	68.612	0	0
12	431.352	0.00	7.20	2.547	−4.4	13.127	2	−0.234	2	61.873	0	1
13	429.398	1.00	6.00	1.6	−5.138	13.313	1	0.393	2	56.434	0	1
14	429.398	1.00	6.00	1.465	−4.885	13.318	2	0.373	2	55.645	0	1
15	476.506	1.00	8.25	1.966	−5.959	10.746	4	0.413	2	56.913	0	2
16	444.394	0.00	7.50	0.503	−4.617	2.887	2	−0.074	1	38.134	0	1
17	458.421	1.00	7.00	1.032	−5.617	2.219	1	0.422	1	39.183	0	1
18	426.403	0.00	7.50	0.327	−4.373	2.814	2	−0.108	1	36.903	0	1
19	488.517	1.00	8.25	2.037	−6.573	7.478	3	0.535	1	54.514	0	2
20	504.517	1.00	10.5	0.638	−3.949	2.767	5	0.053	1	25.635	1	1

Descriptor range or recommended values (according to the QikProp user manual): molecular weight (130.0–725.0), HBD (0.0–6.0), HBA (2.0–20.0), QPlogPo/w predicted octanol/water partition coefficient (–2.0–6.5). QPPCaco (<25 poor, >500 great), Metab (1–8), QPlogKhsa (−1.5–1.5), percent human oral absorption (>80% is high, <25% is poor), human oral absorption (1, 2, or 3 for low, medium, or high.), rule of five (0–4), and rule of three (0–3).

**Table 5:** Predicted cardiotoxicity of all substances.

Compound	Prediction	Binary Reliability %	Multiclass Reliability %	Applicability Domain	IC_50_ Values µm	Reg. Prediction (plC_50_)
**1**	Non-blocker	67.29	35.32	Outside	4.550	5.342
**2**	Non-blocker	87.45	41.2	Outside	12.033	4.92
**3**	Non-blocker	91.46	41.6	Outside	14.613	4.835
**4**	Non-blocker	95.48	46.4	Outside	23.741	4.625
**5**	Non-blocker	76.19	36.9	Outside	15.481	4.81
**6**	Blocker	64.25	34	Outside	7.863	5.104
**7**	Non-blocker	51.86	34.1	Outside	6.416	5.193
**8**	Non-blocker	95.01	31.16	Outside	6.069	5.217
**9**	Non-blocker	96.67	30.52	Outside	4.549	5.342
**10**	Non-blocker	90.21	34.1	Outside	5.679	5.246
**11**	Non-blocker	95.97	33.1	Outside	6.709	5.173
**12**	Non-blocker	77.55	33.44	Outside	7.596	5.119
**13**	Non-blocker	94.07	33.1	Outside	9.50	5.022
**14**	Non-blocker	99.37	32.28	Outside	9.399	5.027
**15**	Non-blocker	99.83	33.58	Outside	10.83	4.965
**16**	Non-blocker	80.06	30.4	Outside	5.490	5.26
**17**	Non-blocker	58.73	28.3	Outside	3.702	5.432
**18**	Blocker	56.83	30.3	Outside	3.275	5.485
**19**	Non-blocker	83.84	34.3	Outside	4.320	5.365
**20**	Non-blocker	98.51	33.41	Outside	7.237	5.14

## Data Availability

The original contributions presented in the study are included in the article and Supplementary Materials, further inquiries can be directed to the corresponding authors.

## Supplementary Materials

The following supporting information can be downloaded at: https://www.mdpi.com/article/10.3390/molecules29194721/s1, Figure S1 Visualization of docking of Ligand 15, 19 docked in protein (6LU7) (a and e). Best binding mode of protein (Ligand 15, 19 as brown and red stick), (b, d, f and h) Amino acid residues involved in bonding interaction (Blue and green dash line represents binding interaction (2D) of ligand 15, 19). Protein with ligand with their best pose (c and g). Figure S2 Visualization of docking of Ligand 20, 18 docked in protein (6LU7) (a and e). Best binding mode of protein (Ligand 20, 18 as brown and red stick), (b, d, f and h) Amino acid residues involved in bonding interaction (Blue and green dash line represents binding interaction (2D) of ligand 20, 18).Protein with ligand with their best pose(c and g). Figure S3 Visualization of docking of Ligand 1, 4 docked in protein (6LU7) (a and e). Best binding mode of protein (Ligand 1, 4 as brown and red stick), (b, d, f and h) Amino acid residues involved in bonding interaction (Blue and green dash line represents binding interaction (2D) of ligand 1, 4).Protein with ligand with their best pose(c and g). Figure S4 Visualization of docking of cyclopropyl Ligand 11, 14 docked in protein (6LU7) (a and e). Best binding mode of protein (Ligand 11, 14 as brown and red stick), (b, d, f and h) Amino acid residues involved in bonding interaction (Blue and green dash line represents binding interaction (2D) of ligand 11, 14).Protein with ligand with their best pose(c and g). Figure S5 Visualization of docking of cyclopropyl Ligand 5, 6 docked in protein (6LU7) (a and e). Best binding mode of protein (Ligand 5, 6 as brown and red stick), (b, d, f and h) Amino acid residues involved in bonding interaction (Blue and green dash line represents binding interaction (2D) of ligand 5, 6).Protein with ligand with their best pose(c and g). Figure S6 Visualization of docking of Ligand 16, 10 docked in protein (6LU7) (a and e). Best binding mode of protein (Ligand 16, 10 as brown and red stick), (b, d, f and h) Amino acid residues involved in bonding interaction (Blue and green dash line represents binding interaction (2D) of ligand 16, 10).Protein with ligand with their best pose(c and g).
