# Supplementary material for: Exploration of Specific Fluoroquinolone Interaction with SARS-CoV-2 Main Protease (Mpro) to Battle COVID-19: DFT, Molecular Docking, ADME and Cardiotoxicity Studies
Source: Molecules. 2024 Oct 5;29(19):4721. doi: 10.3390/molecules29194721 (PMC11477632; doi:10.3390/molecules29194721)
Supplement: Supplementary file 1 [file molecules-29-04721-s001.zip › molecules-3183052-supplementary.pdf]

Supporting File

Exploration of Specific Fluoroquinolone Interaction with SARS-CoV-2 Main Protease (Mpro) to Battle COVID-19: DFT, Molecular Docking, ADME and Cardiotoxicity Studies

Muhammad Asim Khan [1,*], Sadaf Mutahir [1,*], Muhammad Atif Tariq [2] and Abdulrahman A. Almehizia [3]

[1] School of Chemistry and Chemical Engineering, Linyi University, Linyi 276000, China
[2] Department of Chemistry, University of Sialkot, Sialkot 51300, Pakistan; atiftariq380@gmail.com
[3] Department of Pharmaceutical Chemistry, College of Pharmacy, King Saud University, P.O. Box 2457, Riyadh 11451, Saudi Arabia
* Correspondence: khanabdali@lyu.edu.cn (M.A.K.); sadafmutahir@lyu.edu.cn (S.M.)

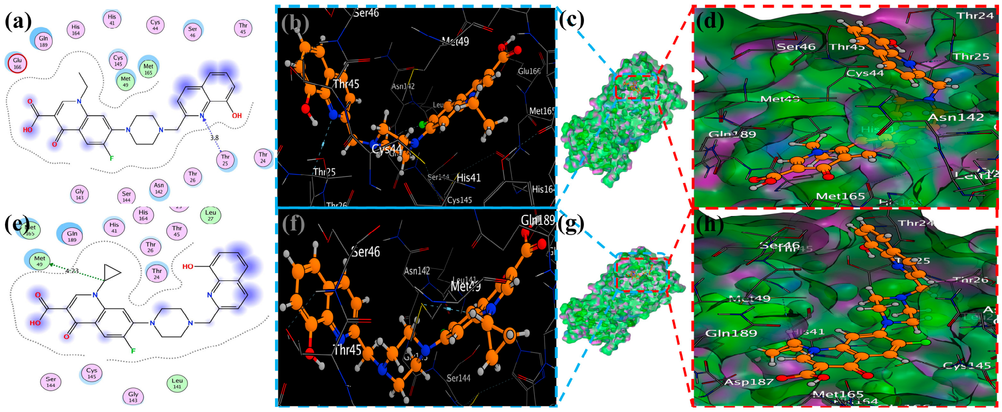

S.1 Visualization of docking of Ligand 15, 19 docked in protein (6LU7) (a and e). Best binding mode of protein (Ligand 15, 19 as brown and red stick), (b, d, f and h) Amino acid residues involved in bonding interaction (Blue and green dash line represents binding interaction (2D) of ligand 15, 19).Protein with ligand with their best pose(c and g).

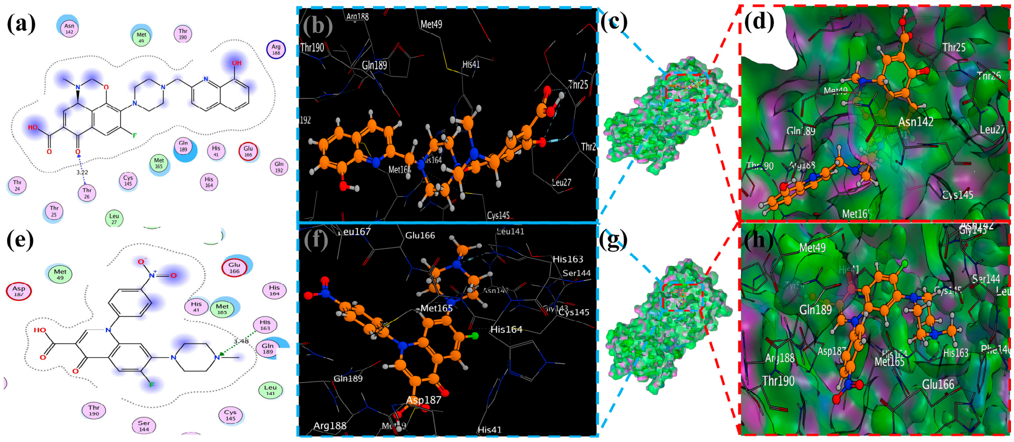

S.2 Visualization of docking of Ligand 20, 18 docked in protein (6LU7) (a and e). Best binding mode of protein (Ligand 20, 18 as brown and red stick), (b, d, f and h) Amino acid residues involved in bonding interaction (Blue and green dash line represents binding interaction (2D) of ligand 20, 18).Protein with ligand with their best pose(c and g).

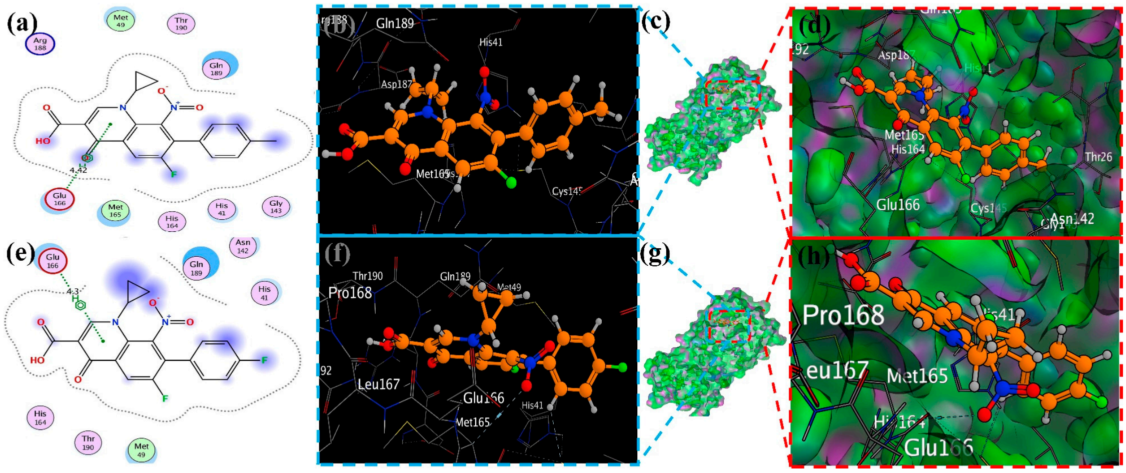

S.3 Visualization of docking of Ligand 1, 4 docked in protein (6LU7) (a and e). Best binding mode of protein (Ligand 1, 4 as brown and red stick), (b, d, f and h) Amino acid residues involved in bonding interaction (Blue and green dash line represents binding interaction (2D) of ligand 1, 4).Protein with ligand with their best pose(c and g).

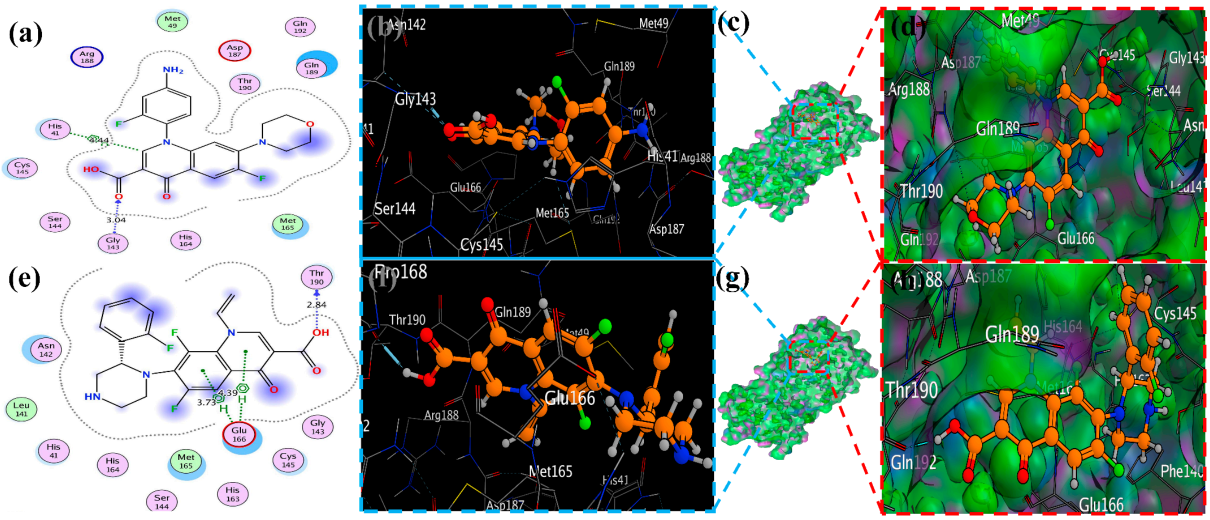

S.4 Visualization of docking of cyclopropyl Ligand 11, 14 docked in protein (6LU7) (a and e). Best binding mode of protein (Ligand 11, 14 as brown and red stick), (b, d, f and h) Amino acid residues involved in bonding interaction (Blue and green dash line represents binding interaction (2D) of ligand 11, 14).Protein with ligand with their best pose(c and g).

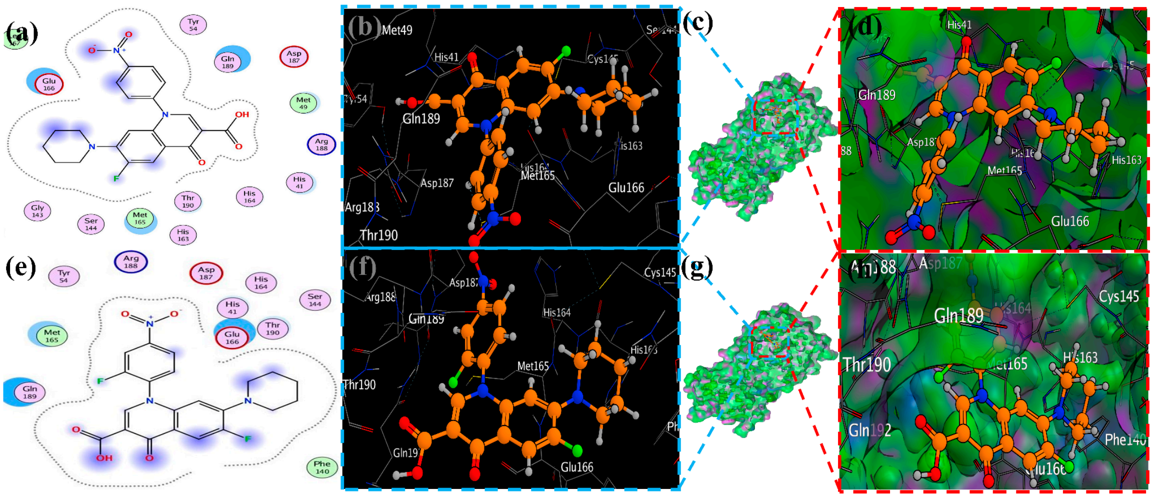

S.5 Visualization of docking of cyclopropyl Ligand 5, 6 docked in protein (6LU7) (a and e). Best binding mode of protein (Ligand 5, 6 as brown and red stick), (b, d, f and h) Amino acid residues involved in bonding interaction (Blue and green dash line represents binding interaction (2D) of ligand 5, 6).Protein with ligand with their best pose(c and g).

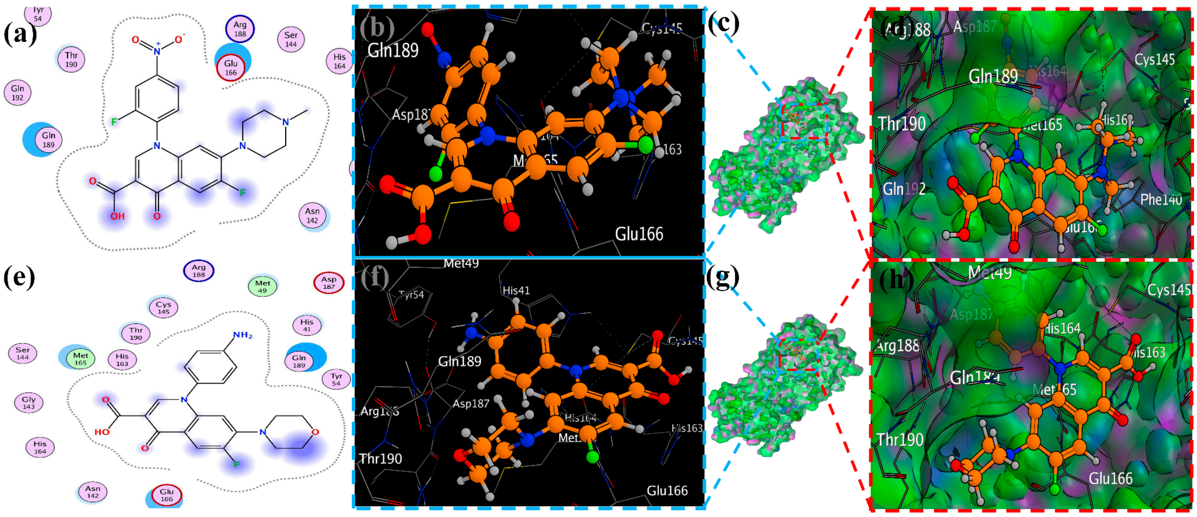

S.6 Visualization of docking of Ligand 16, 10 docked in protein (6LU7) (a and e). Best binding mode of protein (Ligand 16, 10 as brown and red stick), (b, d, f and h) Amino acid residues involved in bonding interaction (Blue and green dash line represents binding interaction (2D) of ligand 16, 10).Protein with ligand with their best pose(c and g).

The geometries of the RCAM-B3LYP/6-311++G (2d, p) optimized clusters in Cartesian-coordinates

Compound 1

$C_{20}H_{15}FN_2O_5$

Center Number	Atomic Number	X	Y	Z
1	7	-0.000009686	0.000000336	0.000008584
2	6	0.000003172	0.000008755	0.000002653
3	6	-0.000004795	-0.000005940	-0.000000403
4	6	-0.000004568	0.000006021	0.000003338

5	6	0.000001079	-0.000015296	0.000000808
6	6	0.000007759	-0.000001282	-0.000001440
7	6	-0.000005703	0.000007047	0.000003446
8	6	-0.000001346	0.000001083	0.000006985
9	6	0.000000499	0.000013400	0.000005006
10	6	0.000009551	0.000018698	-0.000008451
11	6	-0.000005387	-0.000008065	-0.000005478
12	8	0.000009636	0.000008549	0.000002415
13	8	-0.000006138	-0.000004206	-0.000003041
14	8	0.000010272	-0.000000549	-0.000002147
15	7	0.000000907	-0.000006127	0.000000823
16	8	-0.000001676	0.000000162	0.000001343
17	8	-0.000001241	-0.000009140	0.000000550
18	6	-0.000001328	-0.000004602	-0.000021332
19	6	0.000005575	-0.000003335	-0.000005375
20	6	-0.000008292	0.000004557	0.000002151
21	6	-0.000001414	-0.000006132	0.000002258
22	6	0.000001543	0.000002060	0.000001437
23	6	0.000001682	-0.000001093	0.000010308
24	6	0.000000419	0.000004448	-0.000001743
25	9	-0.000001252	-0.000003380	0.000001830
26	6	0.000007750	0.000002388	-0.000016579
27	6	0.000015301	-0.000028526	0.000000039
28	6	-0.000013868	0.000016775	0.000001223
29	1	-0.000000214	-0.000004173	0.000004119
30	1	-0.000003612	-0.000000496	-0.000000161
31	1	0.000001518	0.000001338	0.000006641
32	1	-0.000000384	0.000000003	0.000001637
33	1	-0.000000020	0.000000052	-0.000000847
34	1	-0.000000243	0.000001256	-0.000001436
35	1	0.000002762	0.000002847	0.000004019
36	1	0.000000693	-0.000000440	-0.000000897
37	1	-0.000001235	-0.000001838	-0.000001437
38	1	-0.000000989	-0.000001367	0.000001291
39	1	-0.000003056	-0.000004623	0.000001673
40	1	0.000003091	0.000007386	0.000004843
41	1	-0.000008987	0.000007397	-0.000007564
42	1	0.000004645	-0.000002331	-0.000000244
43	1	-0.000002421	-0.000001616	-0.000000844

Compound 2

$C_{20}H_{15}FN_2O_6$

Center Number	Atomic Number	X	Y	Z
1	7	-0.000004316	-0.000005637	-0.000005526
2	6	0.000004693	-0.000004570	0.000004998
3	6	-0.000002276	0.000007474	-0.000001201
4	6	-0.000011143	0.000013676	0.000000672
5	6	0.000004373	-0.000014165	-0.000000353
6	6	0.000003828	0.000002023	-0.000002872
7	6	-0.000001346	-0.000001605	-0.000000823
8	6	0.000002504	0.000003398	0.000001472
9	6	-0.000006236	-0.000007379	0.000002240
10	6	0.000006722	0.000014728	-0.000000496
11	6	0.000001019	-0.000009246	0.000000548
12	8	0.000002202	0.000008050	-0.000000967
13	8	0.000011025	0.000007209	0.000000996
14	8	-0.000003169	-0.000006434	-0.000002950
15	6	-0.000000319	0.000008113	0.000005746
16	6	-0.000004090	0.000000405	-0.000005034
17	6	-0.000000705	-0.000002160	0.000004677
18	6	0.000006850	0.000006546	-0.000000941
19	6	-0.000001007	-0.000002394	-0.000004751
20	6	0.000002860	-0.000001160	0.000001389
21	9	0.000003596	-0.000003385	0.000002053

22	6	0.000000895	-0.000010261	0.000003837
23	6	-0.000005024	0.000011805	0.000002458
24	6	-0.000002387	-0.000000217	-0.000001923
25	1	-0.000003920	0.000002619	-0.000000642
26	1	-0.000001323	-0.000000690	0.000002274
27	1	-0.000006390	-0.000009776	0.000000962
28	1	-0.000002745	0.000000395	-0.000000058
29	1	-0.000000545	0.000000817	0.000000835
30	1	0.000001664	-0.000000442	0.000001151
31	1	-0.000002551	-0.000001180	-0.000003092
32	1	0.000003009	0.000004197	-0.000005362
33	1	-0.000001885	-0.000001830	-0.000002157
34	1	0.000004562	-0.000003779	0.000001641
35	1	0.000004071	-0.000000410	-0.000000333
36	1	0.000000428	0.000000589	-0.000001141
37	7	0.000000572	0.000006806	0.000012446
38	8	0.000001202	-0.000002194	-0.000009926
39	8	-0.000001575	-0.000005814	0.000000319
40	8	0.000000337	-0.000002492	0.000000373
41	6	-0.000003344	-0.000002847	0.000001352
42	1	-0.000001473	0.000000906	-0.000001622
43	1	-0.000000442	-0.000000618	0.000000611
44	1	0.000001799	0.000000928	-0.000000882

Compound 3

$C_{22}H_{19}FN_2O_6$

Center Number	Atomic Number	X	Y	Z
1	7	0.000011634	-0.000001430	0.000001476
2	6	-0.000006399	-0.000004943	-0.000001498
3	6	0.000001115	0.000004228	-0.000002611
4	6	-0.000001556	-0.000006566	0.000004327
5	6	-0.000000737	0.000007274	-0.000001336
6	6	0.000005905	0.000001106	0.000001682
7	6	-0.000005905	-0.000006968	-0.000004468
8	6	-0.000001624	0.000007526	-0.000000203
9	6	0.000011641	0.000000278	0.000001558
10	6	-0.000009830	-0.000006220	0.000003773
11	6	-0.000001234	0.000003983	-0.000000170
12	8	0.000001558	-0.000005911	0.000003293
13	8	0.000000008	0.000000240	-0.000000362
14	8	0.000006943	0.000003241	-0.000001552
15	6	0.000000768	-0.000001747	-0.000002586
16	6	-0.000005334	-0.000002536	0.000004006
17	6	0.000003107	0.000006771	-0.000004004
18	6	-0.000001875	-0.000000335	0.000000770
19	6	-0.000000501	-0.000000428	0.000003423
20	6	0.000002139	0.000000553	-0.000001726
21	9	0.000001151	0.000003152	0.000002947
22	6	-0.000000387	0.000002452	0.000000782
23	6	0.000000281	-0.000001458	-0.000002579
24	6	-0.000001408	-0.000001126	0.000003443
25	1	0.000000645	0.000000947	-0.000000115
26	1	0.000000121	-0.000000381	-0.000000479
27	1	-0.000003141	0.000006420	-0.000003694
28	1	-0.000000839	-0.000000429	-0.000000301
29	1	0.000000284	-0.000000242	0.000000099
30	1	-0.000000640	-0.000000796	-0.000001622
31	1	-0.000000123	-0.000000219	0.000000229
32	1	-0.000000262	0.000000373	-0.000000310
33	1	0.000001833	0.000000877	-0.000000236
34	1	-0.000000535	-0.000000001	-0.000001128
35	7	-0.000003011	-0.000002026	0.000007603
36	8	-0.000000593	-0.000001482	-0.000006538
37	8	-0.000000619	0.000001569	-0.000002480

38	8	-0.000000395	0.000002909	-0.000003003
39	6	0.000003796	0.000001608	0.000003913
40	1	-0.000001078	-0.000002725	0.000000016
41	1	0.000002202	0.000000178	-0.000000415
42	1	-0.000003460	-0.000005853	0.000000325
43	6	-0.000002595	-0.000005362	-0.000003716
44	1	0.000000883	0.000001131	0.000000008
45	1	0.000001363	0.000004061	0.000000382
46	1	-0.000000277	0.000001439	0.000001707
47	6	-0.000004209	-0.000003515	0.000005824
48	1	-0.000000060	0.000000691	-0.000001376
49	1	0.000002166	0.000000620	0.000000225
50	1	-0.000000916	-0.000000929	-0.000003306

Compound 4

$C_{19}H_{12}F_2N_2O_5$

Center Number	Atomic Number	X	Y	Z
1	7	0.000018340	0.000004135	0.000005771
2	6	-0.000010867	-0.000003958	-0.000005857
3	6	0.000006424	0.000003220	0.000010094
4	6	0.000003431	-0.000005217	-0.000011364
5	6	-0.000005469	-0.000000565	0.000005310
6	6	0.000003666	0.000000822	0.000000984
7	6	-0.000007576	0.000001936	-0.000000047
8	6	0.000002959	-0.000006938	-0.000000849
9	6	0.000011785	0.000002081	-0.000003464
10	6	-0.000012650	-0.000002481	-0.000000639
11	6	-0.000006921	0.000009741	0.000000326
12	8	-0.000002899	-0.000003022	-0.000001063
13	8	0.000023451	0.000002939	0.000003301
14	8	-0.000002939	0.000005576	0.000002438
15	6	-0.000006442	-0.000000732	0.000003178
16	6	0.000004827	-0.000003727	-0.000002355
17	6	-0.000003613	0.000005229	0.000005359
18	9	0.000002944	0.000002754	0.000000932
19	6	-0.000002684	-0.000000494	-0.000008963
20	6	0.000001510	0.000001890	-0.000000673
21	6	-0.000002013	-0.000000715	0.000003366
22	1	0.000001042	0.000000714	-0.000001978
23	1	0.000000978	0.000000486	0.000000277
24	1	-0.000017336	-0.000008743	-0.000003093
25	1	0.000000872	0.000000290	-0.000002422
26	1	-0.000001257	-0.000003848	0.000001262
27	1	0.000001168	-0.000001164	-0.000000441
28	1	-0.000000107	-0.000000760	0.000000257
29	1	-0.000000443	0.000000425	-0.000000115
30	1	0.000000556	-0.000000883	0.000000434
31	1	0.000000411	-0.000000263	0.000000777
32	7	0.000001760	0.000000839	0.000009703
33	8	-0.000002795	0.000001615	-0.000003410
34	8	-0.000003172	-0.000000954	-0.000003370
35	6	-0.000001423	-0.000003105	-0.000005342
36	1	-0.000001128	0.000000652	0.000002310
37	6	-0.000005949	0.000004844	0.000005867
38	6	0.000007712	0.000001390	-0.000004512
39	1	-0.000001040	-0.000001299	-0.000000792
40	9	0.000004886	-0.000002710	-0.000001198

Compound 5

$C_{21}H_{18}FN_3O_5$

Center Number	Atomic Number	X	Y	Z

1	7	-0.000003382	0.000004538	0.000000512
2	6	0.000001194	0.000000752	-0.000000598
3	6	0.000000295	0.000000411	0.000001650
4	6	0.000000105	0.000009973	0.000000157
5	6	0.000001297	-0.000005411	0.000001042
6	6	0.000001047	-0.000000167	0.000000852
7	6	-0.000004372	0.000002120	0.000000441
8	6	-0.000003577	-0.000000926	-0.000005903
9	6	0.000001525	0.000001680	-0.000002899
10	6	0.000002809	0.000000696	0.000003595
11	6	-0.000001536	-0.000010461	-0.000007047
12	8	0.000000772	0.000002361	0.000001585
13	8	0.000006935	0.000004647	0.000005785
14	7	0.000000050	-0.000001846	0.000003892
15	6	0.000001391	-0.000000195	0.000000255
16	6	0.000000443	-0.000000202	0.000000500
17	6	-0.000000408	0.000000638	-0.000000729
18	6	-0.000000215	-0.000000379	0.000000327
19	6	0.000001602	-0.000002490	-0.000000021
20	8	-0.000002024	-0.000007618	-0.000002033
21	6	-0.000004086	-0.000005974	-0.000000096
22	6	0.000001671	-0.000000373	0.000002455
23	6	-0.000000419	-0.000001027	0.000001861
24	6	-0.000002082	0.000002902	0.000000977
25	6	0.000005575	-0.000001932	-0.000002594
26	6	0.000001923	0.000002179	-0.000002292
27	7	-0.000000089	0.000000914	-0.000000945
28	8	-0.000000451	-0.000001275	0.000003488
29	8	-0.000000073	-0.000000227	-0.000003528
30	9	0.000001777	0.000000744	0.000000339
31	1	0.000000410	0.000001112	-0.000001092
32	1	0.000000123	-0.000000117	-0.000000585
33	1	-0.000001657	0.000001598	0.000000797
34	1	-0.000006288	0.000004520	-0.000001044
35	1	-0.000000339	-0.000000062	0.000000143
36	1	-0.000000084	0.000000218	-0.000000257
37	1	0.000000439	-0.000000319	0.000000002
38	1	-0.000000286	-0.000000007	0.000000010
39	1	0.000000249	-0.000000330	-0.000000101
40	1	0.000000112	0.000000028	0.000000236
41	1	0.000000518	-0.000000181	0.000000004
42	1	-0.000000318	-0.000000018	0.000000219
43	1	-0.000000212	0.000000285	-0.000000002
44	1	0.000001592	0.000000474	0.000000103
45	1	-0.000000232	-0.000000397	0.000001827
46	1	0.000000995	-0.000000221	0.000000026
47	1	-0.000001553	0.000000133	-0.000000497
48	1	-0.000001166	-0.000000769	-0.000000818

Compound 6

$C_{21}H_{17}F_2N_3O_5$

Center Number	Atomic Number	X	Y	Z
1	7	0.000013207	-0.000010319	0.000002648
2	6	-0.000017669	0.000014233	0.000013189
3	6	0.000008455	-0.000016224	-0.000002187
4	6	-0.000014313	-0.000024926	-0.000003533
5	6	0.000005726	0.000010935	-0.000003570
6	6	-0.000001762	0.000002337	-0.000001035
7	6	0.000004790	-0.000001267	0.000002795
8	6	-0.000013887	-0.000001162	-0.000000258
9	6	0.000007993	0.000007253	-0.000000070
10	6	-0.000002996	0.000001897	-0.000005485
11	6	-0.000013887	0.000009756	0.000000213

12	8	0.000013897	0.000010917	-0.000002003
13	8	-0.000034234	-0.000006628	0.000011832
14	7	0.000003146	-0.000005088	-0.000004803
15	6	0.000002382	-0.000000561	0.000006613
16	6	-0.000003678	-0.000001521	-0.000002321
17	6	-0.000003331	0.000001480	0.000001539
18	6	-0.000000788	0.000003837	0.000001507
19	6	-0.000000021	0.000000406	0.000001776
20	8	0.000035720	0.000034201	0.000006262
21	6	0.000002689	0.000013786	-0.000009319
22	6	0.000001848	-0.000004039	0.000004932
23	6	-0.000002780	0.000006269	0.000000980
24	6	-0.000009667	-0.000019888	-0.000004462
25	6	0.000001695	0.000001575	-0.000003217
26	6	-0.000003695	-0.000004125	-0.000003864
27	7	0.000031984	0.000001753	0.000008854
28	8	-0.000003420	0.000012972	-0.000012204
29	8	-0.000020622	-0.000002286	0.000012838
30	9	0.000001302	0.000001681	-0.000002181
31	9	-0.000004724	-0.000000988	-0.000001935
32	1	0.000002051	-0.000000396	-0.000002905
33	1	-0.000000251	-0.000001573	0.000001624
34	1	0.000001008	-0.000005219	0.000001287
35	1	0.000011710	-0.000029223	-0.000012014
36	1	0.000000519	-0.000001366	-0.000000913
37	1	-0.000000515	0.000000663	-0.000001000
38	1	0.000000153	-0.000000332	0.000001329
39	1	0.000000291	-0.000000772	0.000000735
40	1	0.000000704	0.000000468	-0.000000522
41	1	0.000000410	0.000000392	-0.000001527
42	1	0.000000119	-0.000000686	0.000000940
43	1	0.000000301	-0.000000137	0.000000310
44	1	-0.000001051	0.000001614	0.000000193
45	1	-0.000000270	-0.000000301	-0.000000702
46	1	-0.000000086	0.000000317	0.000001532
47	1	-0.000000031	0.000000915	-0.000002172
48	1	0.000001577	-0.000000632	0.000000276

Compound 7

$C_{21}H_{20}FN_3O_3$

Center Number	Atomic Number	X	Y	Z
1	7	0.000042549	-0.000012658	-0.000008499
2	6	-0.000068990	-0.000005275	0.000024134
3	6	0.000059998	-0.000022794	-0.000009211
4	6	0.000022993	0.000056395	0.000001560
5	6	-0.000019380	-0.000016753	-0.000000828
6	6	-0.000009378	0.000010164	0.000003999
7	6	-0.000004479	-0.000012596	-0.000006562
8	6	0.000002364	0.000020563	0.000005117
9	6	0.000002994	-0.000007967	0.000001422
10	6	-0.000019066	0.000018999	0.000003686
11	6	-0.000046812	0.000042029	-0.000024842
12	8	0.000021995	-0.000011416	0.000006062
13	8	-0.000002608	-0.000014856	0.000004992
14	7	-0.000003729	-0.000023045	-0.000002167
15	6	-0.000000240	0.000007756	0.000000184
16	6	-0.000002694	-0.000001640	-0.000004220
17	6	0.000005589	0.000000409	0.000003677
18	6	-0.000001605	-0.000006986	-0.000004560
19	6	0.000002127	0.000013931	0.000002958
20	8	-0.000013331	-0.000020590	-0.000005171
21	9	0.000002912	0.000005987	0.000002508
22	6	-0.000000411	0.000006599	-0.000005407
23	6	0.000000213	-0.000002464	-0.000000214

24	6	0.000002815	0.000007309	0.000000263
25	6	-0.000009664	-0.000001770	0.000000984
26	6	0.000005285	0.000005908	-0.000001177
27	6	-0.000000473	-0.000002269	0.000000160
28	7	0.000033869	-0.000001215	-0.000004855
29	1	0.000020632	0.000005686	0.000000651
30	1	0.000003498	-0.000005977	0.000000064
31	1	-0.000004332	-0.000000002	-0.000000977
32	1	0.000007469	-0.000020613	0.000010473
33	1	-0.000000443	-0.000002932	0.000001774
34	1	0.000000408	-0.000003972	-0.000001479
35	1	-0.000001948	0.000001067	0.000001825
36	1	-0.000000550	-0.000000060	0.000001967
37	1	-0.000000969	0.000003186	0.000000457
38	1	-0.000000137	-0.000001486	-0.000001264
39	1	0.000002775	0.000000950	0.000000478
40	1	0.000005846	-0.000000482	-0.000000340
41	1	-0.000005163	-0.000000471	0.000001087
42	1	0.000000302	-0.000000919	-0.000000545
43	1	0.000000477	0.000000760	0.000000334
44	1	-0.000001474	-0.000000209	-0.000000436
45	1	-0.000001553	-0.000000187	0.000000181
46	1	0.000000290	0.000000225	-0.000000365
47	1	-0.000014823	-0.000002872	-0.000000685
48	1	-0.000013151	-0.000003448	0.000002806

Compound 8

$C_{21}H_{19}F_2N_3O_3$

Center Number	Atomic Number	X	Y	Z
1	6	-0.000007769	0.000004607	0.000003717
2	6	-0.000002670	-0.000000420	0.000000485
3	6	0.000006771	0.000005503	0.000000807
4	6	0.000004914	-0.000003851	0.000001425
5	6	-0.000007187	0.000002117	-0.000001782
6	6	0.000017107	-0.000001661	0.000000195
7	1	0.000000432	-0.000001044	0.000000207
8	6	-0.000008537	-0.000026480	-0.000006050
9	1	-0.000000172	0.000001043	-0.000000153
10	6	0.000002857	-0.000006791	0.000000400
11	6	0.000002762	0.000008680	0.000001233
12	1	-0.000001466	0.000001177	-0.000000719
13	6	-0.000001994	-0.000010137	-0.000002318
14	6	-0.000000602	-0.000002809	0.000002672
15	6	0.000003532	0.000000447	0.000008521
16	6	0.000000742	0.000007101	-0.000001677
17	1	-0.000000042	0.000000030	-0.000001253
18	6	-0.000001012	0.000001412	0.000001957
19	6	0.000004638	0.000004445	-0.000005087
20	1	0.000000140	-0.000001561	-0.000000798
21	1	0.000000386	-0.000000834	0.000000626
22	6	-0.000020865	0.000007638	0.000001787
23	7	-0.000019518	0.000003559	0.000002665
24	1	0.000006630	-0.000004832	0.000000849
25	1	0.000006675	-0.000004713	-0.000001673
26	9	-0.000000643	0.000001940	-0.000002123
27	7	-0.000001189	0.000014496	-0.000004064
28	7	-0.000009995	0.000002967	-0.000000581
29	9	0.000003455	-0.000006963	-0.000003896
30	8	0.000004925	0.000011243	0.000002170
31	8	0.000005977	0.000001278	-0.000000750
32	8	0.000008621	-0.000011907	-0.000000647
33	1	-0.000002255	0.000002040	-0.000000168
34	6	0.000004446	-0.000001193	-0.000002537
35	1	0.000001024	0.000002191	0.000001904

36	1	-0.000001197	0.000001275	0.000002466
37	6	-0.000002279	0.000001283	0.000001434
38	1	0.000001182	-0.000001569	-0.000000903
39	1	0.000000037	-0.000000404	-0.000000729
40	6	0.000003691	-0.000004415	0.000001281
41	1	-0.000002190	0.000001625	0.000001306
42	1	0.000000374	0.000001541	-0.000000606
43	6	-0.000001028	0.000000138	0.000002672
44	1	0.000000202	-0.000000166	-0.000000562
45	1	0.000000454	-0.000000130	-0.000001984
46	6	-0.000000512	-0.000000576	-0.000001800
47	1	0.000000385	0.000001419	0.000000424
48	1	0.000000760	0.000001263	0.000001657

Compound 9

$C_{20}H_{16}FN_3O_6$

Center Number	Atomic Number	X	Y	Z
1	7	-0.000002097	0.000002726	-0.000001310
2	6	0.000001191	-0.000001221	0.000001861
3	6	0.000001621	-0.000001035	0.000002446
4	6	-0.000003507	0.000008272	0.000000764
5	6	0.000000631	-0.000004730	-0.000000071
6	6	-0.000001272	-0.000001031	0.000000169
7	6	-0.000001309	-0.000002164	-0.000000289
8	6	0.000002092	0.000005421	-0.000002044
9	6	-0.000005328	-0.000003912	0.000001240
10	6	0.000002439	0.000002992	-0.000001205
11	6	-0.000001041	-0.000002455	-0.000004497
12	8	-0.000000749	-0.000001087	0.000001402
13	8	0.000005593	0.000008313	0.000000240
14	8	0.000001924	-0.000002991	-0.000000629
15	6	0.000005939	-0.000003454	0.000001623
16	6	-0.000000874	0.000006505	-0.000000138
17	6	0.000001488	-0.000004356	-0.000002131
18	6	-0.000002120	-0.000010208	0.000002437
19	6	-0.000000142	0.000001800	-0.000003317
20	6	-0.000001632	0.000003121	-0.000001017
21	7	0.000000570	0.000007214	-0.000000581
22	8	0.000001070	-0.000002176	0.000002381
23	8	-0.000000916	-0.000000699	-0.000001437
24	7	0.000001484	-0.000002853	0.000006071
25	6	0.000000165	0.000002601	-0.000003555
26	6	0.000001972	0.000002547	0.000003191
27	8	-0.000000308	-0.000001226	-0.000002203
28	6	-0.000002320	-0.000004501	-0.000000005
29	6	0.000002544	-0.000000689	-0.000001148
30	9	0.000003057	0.000000156	-0.000000708
31	1	-0.000000953	0.000000318	0.000000517
32	1	0.000000672	0.000000238	-0.000000548
33	1	-0.000001267	0.000001068	0.000001090
34	1	-0.000005906	-0.000005865	0.000002330
35	1	-0.000000641	-0.000000216	0.000000609
36	1	-0.000000158	0.000000549	0.000000692
37	1	0.000000772	-0.000000122	-0.000000397
38	1	-0.000000031	0.000001139	0.000000027
39	1	-0.000000349	0.000001245	0.000002054
40	1	-0.000001058	0.000000625	-0.000002065
41	1	-0.000000549	0.000000139	0.000000697
42	1	-0.000000308	0.000000644	-0.000000201
43	1	-0.000000135	-0.000000329	0.000000180
44	1	-0.000000919	0.000000735	-0.000000590
45	1	0.000000605	-0.000000508	-0.000000726
46	1	0.000000061	-0.000000539	-0.000001209

Compound 10

$C_{20}H_{18}FN_3O_4$

Center Number	Atomic Number	X	Y	Z
1	7	-0.000006367	0.000002104	0.000004748
2	6	-0.000001105	-0.000002443	-0.000001493
3	6	0.000018025	0.000007931	-0.000001575
4	6	0.000006202	0.000005956	0.000003284
5	6	-0.000003239	-0.000001395	-0.000001110
6	6	0.000000718	0.000000274	-0.000000688
7	6	-0.000007028	-0.000000290	-0.000001501
8	6	0.000010773	0.000004569	0.000002727
9	6	0.000002135	-0.000002037	0.000001624
10	6	0.000002438	-0.000002228	-0.000002519
11	6	-0.000012452	-0.000015412	0.000003467
12	8	-0.000000204	0.000006106	-0.000000833
13	8	-0.000041465	0.000027086	-0.000004498
14	8	-0.000001904	-0.000002077	-0.000003252
15	7	-0.000011427	-0.000001923	-0.000002871
16	6	0.000004150	-0.000000425	0.000000034
17	6	-0.000000650	0.000001748	-0.000000741
18	8	-0.000001382	0.000000173	0.000000044
19	6	0.000001697	-0.000001097	0.000000595
20	6	0.000001206	0.000001438	0.000001298
21	9	-0.000000598	-0.000002430	-0.000000849
22	6	0.000002507	-0.000001294	-0.000003014
23	6	-0.000000967	-0.000003722	0.000005027
24	6	0.000003028	0.000005408	0.000001813
25	6	-0.000000593	0.000000575	-0.000009272
26	6	-0.000002903	-0.000003792	0.000004884
27	6	0.000000387	0.000003963	-0.000001865
28	7	0.000002754	-0.000001879	0.000006029
29	1	-0.000000753	-0.000001183	-0.000000377
30	1	0.000001901	-0.000000514	-0.000000417
31	1	-0.000000350	0.000001019	-0.000000450
32	1	0.000040893	-0.000024272	0.000004255
33	1	-0.000000568	0.000001982	-0.000000026
34	1	0.000001153	-0.000000007	0.000000942
35	1	0.000000547	-0.000000632	0.000000034
36	1	0.000000200	0.000000598	-0.000000599
37	1	0.000000233	0.000000718	-0.000000201
38	1	-0.000000419	0.000000199	-0.000000174
39	1	0.000000727	-0.000000355	-0.000000159
40	1	-0.000002045	0.000000011	-0.000000525
41	1	-0.000000726	0.000000325	0.000000309
42	1	-0.000000128	-0.000001513	0.000000475
43	1	-0.000000777	0.000000151	-0.000000990
44	1	-0.000000210	-0.000001013	-0.000000317
45	1	0.000001045	-0.000000587	0.000000357
46	1	-0.000004456	0.000000190	-0.000001632

Compound 11

$C_{20}H_{17}F_2N_3O_4$

Center Number	Atomic Number	X	Y	Z
1	6	-0.000004166	-0.000002790	-0.000003528
2	6	0.000003759	0.000004165	0.000002742
3	6	0.000000756	-0.000005310	0.000004308
4	6	0.000001561	0.000005103	-0.000003258
5	6	0.000001917	-0.000002534	-0.000000243
6	6	0.000003072	0.000000782	-0.000001460
7	1	-0.000000183	-0.000000434	-0.000000483

8	6	-0.000003011	0.000001941	-0.000003917
9	1	-0.000002883	0.000000402	0.000000677
10	6	0.000003982	0.000004520	-0.000000794
11	6	0.000005454	-0.000007030	0.000001533
12	1	-0.000000064	0.000000391	-0.000000097
13	6	-0.000001716	-0.000002053	0.000008517
14	6	0.000001969	-0.000003090	-0.000004008
15	6	-0.000000619	0.000006061	-0.000001758
16	6	-0.000001531	0.000000584	-0.000002582
17	1	0.000000772	-0.000000978	0.000001298
18	6	-0.000000281	-0.000000706	-0.000001014
19	6	0.000003497	0.000002795	0.000004275
20	1	-0.000001527	0.000001222	0.000000405
21	1	0.000000308	-0.000000613	-0.000000204
22	6	-0.000002839	0.000003360	-0.000000723
23	7	0.000000387	0.000001839	-0.000002062
24	1	-0.000000048	-0.000001879	0.000000495
25	1	-0.000000322	-0.000002279	0.000001300
26	9	0.000000454	-0.000003257	-0.000002202
27	7	-0.000004266	-0.000000337	0.000000859
28	7	-0.000005128	-0.000002382	-0.000000216
29	9	0.000000028	0.000001767	0.000000902
30	8	0.000001167	-0.000000663	0.000000896
31	8	-0.000001178	0.000002110	0.000001072
32	8	-0.000004157	-0.000005035	0.000000069
33	1	0.000002801	-0.000000097	0.000000146
34	6	0.000003008	0.000002538	0.000002009
35	1	0.000000861	-0.000000613	0.000000110
36	1	-0.000000432	0.000000774	-0.000000894
37	6	-0.000000116	-0.000004844	-0.000000620
38	1	0.000000291	0.000001405	-0.000000051
39	1	-0.000000573	0.000000676	-0.000000606
40	6	0.000001593	0.000000187	0.000001898
41	1	-0.000002401	0.000001324	-0.000000837
42	1	0.000001590	-0.000000662	-0.000001246
43	6	-0.000000349	0.000000490	0.000002427
44	1	-0.000000623	-0.000000070	-0.000000747
45	1	0.000000179	-0.000000050	-0.000000460
46	8	-0.000000993	0.000003269	-0.000001929

Compound 12

$C_{20}H_{15}F_2N_3O_6$

Center Number	Atomic Number	X	Y	Z
1	7	0.000005277	0.000009091	0.000001245
2	6	-0.000002254	-0.000003980	-0.000001745
3	6	0.000002415	0.000002848	0.000002023
4	6	0.000009111	0.000006248	0.000002230
5	6	-0.000003696	-0.000006093	-0.000001813
6	6	0.000001582	0.000000438	0.000001727
7	6	0.000000382	-0.000005816	-0.000001987
8	6	0.000004689	0.000011921	0.000008554
9	6	0.000000184	-0.000000468	0.000000076
10	6	-0.000008527	-0.000004723	-0.000002205
11	8	-0.000000371	0.000000296	0.000000805
12	8	0.000007432	-0.000004454	-0.000004016
13	8	-0.000006062	-0.000001365	0.000000916
14	7	0.000002665	-0.000011150	-0.000013844
15	6	-0.000006616	0.000006065	0.000006475
16	6	0.000005398	0.000002185	-0.000000110
17	8	-0.000002203	-0.000002202	0.000001392
18	6	0.000001902	-0.000000012	-0.000000957
19	6	0.000000061	-0.000001838	0.000002750
20	6	-0.000006171	-0.000002288	0.000000042
21	6	-0.000001463	-0.000001320	0.000000751

22	6	0.000001712	0.000000278	0.000000943
23	6	0.000005543	0.000000909	-0.000003469
24	6	-0.000005822	-0.000001152	-0.000002906
25	6	0.000004200	0.000003551	0.000002100
26	7	-0.000006956	-0.000001872	0.000004453
27	8	0.000002852	0.000001432	-0.000003218
28	8	0.000002038	0.000000563	-0.000003575
29	9	-0.000001305	0.000003617	0.000002100
30	1	-0.000000901	0.000000416	0.000001127
31	1	-0.000000657	0.000001352	0.000001498
32	1	-0.000003963	0.000003572	0.000004090
33	1	0.000000367	-0.000002469	0.000001990
34	1	0.000000372	-0.000002271	-0.000001081
35	1	-0.000000410	-0.000000673	-0.000000843
36	1	0.000000528	0.000000286	0.000000851
37	1	0.000000130	-0.000000206	-0.000000229
38	1	0.000000376	0.000000154	-0.000000038
39	1	-0.000000888	0.000002457	-0.000002245
40	1	0.000000342	0.000000275	0.000000788
41	1	-0.000001805	-0.000000015	-0.000001342
42	1	0.000000461	-0.000000039	-0.000000583
43	6	-0.000000870	-0.000002241	-0.000001160
44	1	-0.000000395	-0.000000106	-0.000000924
45	1	-0.000000282	-0.000000037	-0.000000435
46	9	0.000001603	-0.000001164	-0.000000201

Compound 13

$C_{22}H_{18}F_3N_3O_3$

Center Number	Atomic Number	X	Y	Z
1	7	0.000000530	-0.000004039	0.000000722
2	6	-0.000002720	0.000001209	-0.000002635
3	6	0.000003820	-0.000001372	0.000004910
4	6	0.000003242	-0.000015381	-0.000010646
5	6	-0.000001458	0.000001397	-0.000000864
6	6	0.000010078	0.000003037	0.000003051
7	6	-0.000008225	0.000006760	0.000008205
8	6	-0.000001937	-0.000010128	-0.000006469
9	6	0.000014196	-0.000001602	-0.000000308
10	6	-0.000006258	0.000005614	0.000002507
11	6	-0.000014299	0.000006567	0.000005479
12	8	0.000002503	0.000000110	-0.000001095
13	8	0.000007580	-0.000007645	-0.000003756
14	8	-0.000005258	0.000011630	0.000008507
15	7	0.000000095	-0.000004705	0.000002111
16	6	0.000007369	-0.000000513	0.000000897
17	6	-0.000004359	0.000003315	-0.000001063
18	7	0.000003413	-0.000000823	0.000000069
19	6	-0.000001063	-0.000000131	0.000000667
20	6	-0.000000893	0.000006604	-0.000006075
21	6	0.000000736	0.000007229	0.000003754
22	6	-0.000000504	-0.000001767	-0.000001621
23	6	-0.000000701	-0.000001187	-0.000000626
24	6	0.000000389	0.000001036	0.000000662
25	6	-0.000000571	0.000003373	-0.000001432
26	6	-0.000000551	-0.000007897	0.000000899
27	9	-0.000000568	-0.000001593	0.000000436
28	9	-0.000002137	0.000000584	-0.000002529
29	6	-0.000002356	0.000001888	0.000001731
30	6	0.000001573	0.000000128	-0.000001518
31	1	0.000001402	0.000001784	-0.000000786
32	1	-0.000003414	-0.000001003	0.000000085
33	1	0.000000046	0.000001442	0.000001238
34	1	-0.000000121	-0.000001125	-0.000001499

35	1	0.000000456	-0.000000036	-0.000001114
36	1	0.000000899	-0.000000254	-0.000000614
37	1	-0.000000992	0.000000312	0.000000660
38	1	-0.000000542	0.000000059	-0.000000897
39	1	0.000001014	0.000000496	-0.000000321
40	1	-0.000000137	-0.000000643	0.000001333
41	1	-0.000000272	-0.000000768	0.000000460
42	1	0.000000037	0.000002775	-0.000000605
43	1	-0.000000497	0.000000033	-0.000000507
44	1	0.000000219	0.000000514	0.000000289
45	1	0.000000113	-0.000002626	-0.000001748
46	1	0.000000215	-0.000000015	-0.000000361
47	1	0.000000302	-0.000000636	-0.000000059
48	1	0.000000066	-0.000001477	0.000000171
49	9	-0.000000463	-0.000000532	0.000000306

Compound 14

$C_{21}H_{18}F_2N_4O_5$

Center Number	Atomic Number	X	Y	Z
1	6	0.000006578	0.000010056	-0.000001455
2	6	0.000005431	0.000001813	-0.000000536
3	6	-0.000005464	0.000002832	-0.000001261
4	6	-0.000008038	-0.000003942	-0.000000364
5	6	0.000007139	-0.000001836	0.000000583
6	6	-0.000012722	-0.000003142	-0.000001178
7	1	-0.000001587	-0.000000504	-0.000000143
8	6	0.000007511	-0.000016243	0.000001053
9	1	0.000000986	0.000000487	0.000000090
10	6	-0.000003160	-0.000008698	-0.000000474
11	6	-0.000005093	0.000008467	-0.000000667
12	1	0.000001190	0.000000601	0.000000279
13	6	0.000002295	-0.000014443	0.000004053
14	6	-0.000003026	0.000008750	-0.000008044
15	6	-0.000001566	0.000002951	-0.000003201
16	6	0.000002143	-0.000014317	0.000004410
17	6	-0.000000309	-0.000006467	-0.000002393
18	1	0.000000277	0.000000848	0.000000934
19	6	-0.000000454	0.000015349	0.000001960
20	1	-0.000000434	0.000001236	-0.000001023
21	1	0.000000390	0.000000015	0.000001518
22	7	-0.000000937	-0.000006523	0.000001033
23	8	0.000000315	0.000002135	0.000000578
24	8	-0.000000436	0.000000659	-0.000001655
25	9	0.000000372	0.000001843	0.000002315
26	9	-0.000004773	-0.000005183	-0.000000064
27	7	0.000009721	0.000000009	0.000005476
28	7	-0.000000936	0.000002816	-0.000002026
29	7	0.000002652	0.000018016	0.000002239
30	6	-0.000000722	-0.000002175	0.000003206
31	1	0.000000869	-0.000000296	-0.000000565
32	1	0.000000461	-0.000000257	-0.000000785
33	1	0.000000676	0.000000387	-0.000000594
34	8	-0.000003683	0.000007621	0.000000196
35	6	0.000019579	0.000020316	0.000002080
36	8	-0.000002184	-0.000002800	-0.000000923
37	8	-0.000010836	-0.000018545	-0.000001833
38	1	0.000001918	0.000002511	0.000000291
39	6	0.000004914	-0.000001792	0.000000667
40	1	0.000000669	0.000000227	-0.000000079
41	1	-0.000001425	-0.000000555	0.000000536
42	6	-0.000007830	-0.000000804	-0.000003568
43	1	0.000000627	-0.000000076	0.000000920
44	1	0.000001276	0.000000328	-0.000000147
45	6	-0.000005397	0.000000327	-0.000001817

46	1	0.000001439	0.000000014	-0.000000206
47	1	-0.000000706	0.000000387	0.000000860
48	6	0.000002778	-0.000001810	-0.000000894
49	1	-0.000000757	-0.000000233	0.000001195
50	1	0.000000268	-0.000000360	-0.000000577

Compound 15

$C_{26}H_{25}FN_4O_4$

Center Number	Atomic Number	X	Y	Z
1	7	0.000019147	-0.000003494	-0.000000960
2	6	-0.000016808	-0.000006178	-0.000001593
3	6	0.000002258	0.000006431	-0.000000580
4	6	0.000008248	-0.000003949	0.000001221
5	6	-0.000003659	-0.000002454	0.000000193
6	6	0.000000961	0.000000112	-0.000000196
7	6	-0.000000686	-0.000001385	-0.000000860
8	6	-0.000001675	0.000002343	-0.000000369
9	6	0.000002118	-0.000002362	-0.000000553
10	6	-0.000009074	0.000010463	-0.000000703
11	6	-0.000003332	0.000002447	0.000000542
12	8	0.000000286	-0.000000311	-0.000000836
13	8	0.000000717	-0.000001106	0.000000048
14	8	0.000000033	0.000001299	0.000000165
15	7	0.000000455	-0.000000905	0.000001620
16	6	0.000000420	0.000000066	-0.000001729
17	6	0.000000074	-0.000001320	0.000000186
18	7	-0.000000342	0.000000599	-0.000001511
19	6	-0.000000227	-0.000000170	0.000001168
20	6	-0.000000197	0.000001582	-0.000000072
21	6	0.000001412	0.000000100	0.000001090
22	7	0.000000021	-0.000001106	0.000000957
23	6	-0.000000676	-0.000000011	0.000000231
24	6	0.000000365	0.000000305	-0.000000171
25	6	-0.000000285	0.000000028	-0.000000288
26	6	0.000000128	0.000000033	-0.000000519
27	6	-0.000000840	0.000000291	0.000000113
28	6	0.000000636	-0.000000859	0.000000481
29	6	0.000000583	-0.000000616	0.000000067
30	6	-0.000001071	0.000000594	-0.000000336
31	6	0.000000453	-0.000000845	-0.000000137
32	8	0.000000479	-0.000001124	0.000000887
33	9	-0.000000007	-0.000000606	0.000000347
34	6	-0.000001170	-0.000002110	0.000002474
35	6	-0.000000594	0.000001027	-0.000000218
36	1	0.000003310	0.000000671	0.000000005
37	1	-0.000000117	-0.000000056	0.000000106
38	1	-0.000000041	0.000001293	0.000000240
39	1	-0.000002783	-0.000000016	-0.000000736
40	1	0.000000033	0.000000098	0.000000127
41	1	-0.000000118	0.000000073	0.000000012
42	1	0.000000053	0.000000082	0.000000045
43	1	-0.000000078	-0.000000045	-0.000000013
44	1	0.000000243	-0.000000124	-0.000000036
45	1	-0.000000020	-0.000000156	-0.000000105
46	1	0.000000247	-0.000000186	0.000000126
47	1	-0.000000219	-0.000000148	-0.000000114
48	1	-0.000000509	-0.000000044	-0.000000238
49	1	0.000000054	-0.000000048	0.000000076
50	1	-0.000000242	-0.000000155	0.000000239
51	1	0.000000114	-0.000000059	0.000000087
52	1	0.000000247	-0.000000243	-0.000000060
53	1	-0.000000042	-0.000000230	-0.000000096
54	1	-0.000000004	-0.000000391	-0.000000042
55	1	-0.000000223	0.000000015	-0.000000365

56	1	0.000000704	0.000000605	0.000000453
57	1	-0.000000003	0.000000577	0.000000145
58	1	0.000000456	0.000000665	-0.000000015
59	1	0.000000120	0.000000203	-0.000000106
60	1	0.000000667	0.000000810	0.000000108

Compound 16

$C_{22}H_{18}F_3N_3O_3$

Center Number	Atomic Number	X	Y	Z
1	7	-0.000032772	-0.000002865	-0.000018808
2	6	0.000014061	-0.000004092	0.000005264
3	6	0.000003487	-0.000001272	-0.000032374
4	6	-0.000019548	0.000010172	0.000017524
5	6	0.000003735	0.000011248	0.000006093
6	6	0.000000477	-0.000004877	-0.000008663
7	6	0.000001039	-0.000001505	-0.000002088
8	6	0.000029969	-0.000016446	-0.000007979
9	6	-0.000027510	0.000003373	0.000004660
10	6	0.000027986	0.000003941	0.000015859
11	6	-0.000004220	-0.000018759	0.000016791
12	8	0.000007571	0.000013912	0.000007905
13	8	-0.000007614	0.000009000	0.000011666
14	8	0.000020160	-0.000007177	0.000012940
15	7	-0.000009721	0.000026629	0.000031454
16	6	-0.000014504	-0.000019285	-0.000001420
17	6	-0.000003139	0.000006091	-0.000001455
18	7	0.000003141	0.000001277	-0.000000456
19	6	-0.000006355	-0.000001662	0.000002182
20	6	0.000002507	0.000004808	-0.000020100
21	6	-0.000001836	0.000005830	-0.000004883
22	6	-0.000007943	-0.000003756	0.000002979
23	6	0.000002980	0.000002595	0.000002558
24	6	-0.000000958	-0.000004084	-0.000000025
25	6	-0.000000064	-0.000003697	0.000000519
26	6	0.000002105	-0.000000546	-0.000005964
27	9	0.000006698	0.000004316	0.000000644
28	9	0.000004476	-0.000000966	-0.000004765
29	9	0.000002580	-0.000000589	-0.000003670
30	6	-0.000004907	-0.000004854	0.000008865
31	6	0.000002879	0.000010565	0.000002886
32	1	-0.000001105	-0.000007454	-0.000000743
33	1	0.000000960	0.000000965	0.000000158
34	1	0.000004402	-0.000018697	-0.000038270
35	1	0.000000073	0.000002571	0.000001785
36	1	-0.000001811	-0.000000203	-0.000001587
37	1	-0.000000126	-0.000001583	-0.000000464
38	1	-0.000001767	0.000000484	-0.000002327
39	1	0.000000307	0.000000217	-0.000001192
40	1	0.000002064	0.000002740	0.000001833
41	1	0.000002150	-0.000000811	-0.000000668
42	1	0.000002475	0.000000357	0.000001451
43	1	-0.000000539	-0.000000013	0.000000266
44	1	-0.000000117	-0.000000183	-0.000000213
45	1	-0.000000907	-0.000000322	-0.000000993
46	1	-0.000000122	-0.000000129	-0.000000151
47	1	0.000000342	0.000002266	0.000001789
48	1	-0.000001003	0.000001589	0.000000267
49	1	-0.000000034	0.000000882	0.000000918

Compound 17

$C_{22}H_{18}F_2N_4O_5$

Center Atomic

Number	Number	X	Y	Z
1	6	0.000000774	-0.000000438	0.000000913
2	6	-0.000000465	-0.000000593	-0.000000092
3	6	-0.000000259	0.000000836	0.000000687
4	6	0.000000952	-0.000000479	0.000000013
5	6	-0.000002054	0.000000027	-0.000000330
6	6	0.000000616	-0.000000226	0.000001170
7	1	0.000000067	0.000000020	0.000000063
8	6	0.000000369	-0.000001376	-0.000003854
9	1	-0.000000566	0.000000731	-0.000000033
10	6	0.000000420	-0.000000590	-0.000000346
11	6	0.000000426	0.000001304	0.000001466
12	1	0.000000210	0.000000197	-0.000000121
13	6	0.000000021	0.000000425	-0.000000520
14	6	0.000000326	-0.000000360	-0.000000128
15	6	-0.000000364	-0.000000004	-0.000000127
16	6	-0.000000267	0.000001284	-0.000000086
17	6	-0.000000031	0.000001105	0.000000167
18	1	0.000000060	-0.000000022	-0.000000059
19	6	-0.000000231	-0.000002144	-0.000000097
20	1	-0.000000115	0.000000140	-0.000000018
21	1	-0.000000080	0.000000113	0.000000028
22	7	0.000000659	0.000001651	-0.000000199
23	8	-0.000000640	-0.000000791	-0.000001463
24	8	-0.000000050	-0.000000763	0.000001720
25	9	-0.000000091	0.000000130	0.000000162
26	9	-0.000000362	-0.000000035	-0.000000465
27	7	-0.000004478	0.000000201	-0.000007880
28	7	0.000003471	-0.000002880	0.000001120
29	7	-0.000000315	-0.000000216	0.000001568
30	8	-0.000000266	0.000000804	0.000000787
31	6	0.000000189	-0.000001075	-0.000001660
32	8	-0.000000268	0.000000084	0.000001202
33	8	-0.000000109	0.000001422	-0.000000421
34	1	-0.000000328	-0.000000026	0.000000080
35	6	-0.000004741	0.000000943	-0.000001401
36	6	0.000008669	-0.000001586	0.000004545
37	1	0.000000027	-0.000000302	0.000000573
38	1	-0.000002061	0.000000574	-0.000000320
39	6	0.000003379	-0.000000192	0.000002723
40	1	-0.000001360	-0.000000502	0.000000164
41	1	0.000000425	-0.000000342	0.000000150
42	6	-0.000001536	0.000001502	-0.000000216
43	1	-0.000000982	0.000001153	0.000000859
44	6	-0.000000525	0.000001190	-0.000002025
45	1	0.000000183	-0.000000883	0.000000451
46	1	-0.000000207	-0.000000247	0.000000446
47	1	-0.000000079	-0.000000244	0.000000524
48	6	0.000001103	-0.000000439	0.000000234
49	1	-0.000000238	-0.000000005	-0.000000131
50	1	-0.000000242	0.000000222	-0.000000096
51	1	0.000000965	0.000000702	0.000000278

Compound 18

$C_{21}H_{19}FN_4O_5$

Center Number	Atomic Number	Forces (Hartrees/Bohr) X	Y	Z
1	7	-0.000000751	-0.000000786	-0.000006911
2	6	0.000005821	0.000004422	-0.000003326
3	6	-0.000004005	-0.000002936	-0.000007538
4	6	0.000005201	0.000014596	0.000002107
5	6	-0.000001565	-0.000002771	0.000003995
6	6	0.000000864	0.000000522	0.000001232

7	6	-0.000002118	-0.000001080	-0.000005493
8	6	0.000008609	0.000001640	-0.000001232
9	6	-0.000000204	-0.000001026	-0.000000150
10	6	0.000001160	0.000004227	0.000000433
11	6	0.000010067	-0.000010559	0.000017149
12	8	-0.000007293	0.000005458	-0.000005240
13	8	-0.000001204	0.000004624	-0.000005932
14	8	-0.000004177	-0.000010995	-0.000002320
15	6	-0.000000146	-0.000003538	0.000002619
16	6	-0.000000271	0.000004689	0.000000977
17	6	0.000000143	0.000000417	-0.000001255
18	6	-0.000001319	0.000001634	-0.000002185
19	6	0.000000402	0.000001911	0.000001015
20	6	0.000000164	-0.000001866	-0.000001268
21	7	0.000004692	-0.000000187	0.000001288
22	8	-0.000002658	-0.000003628	0.000001242
23	8	-0.000001961	-0.000003261	-0.000002512
24	7	-0.000007299	0.000000494	0.000005048
25	6	0.000000646	0.000000221	-0.000003249
26	6	0.000000276	-0.000001020	0.000000735
27	7	-0.000000727	0.000000604	0.000000676
28	6	0.000000483	-0.000000525	-0.000000435
29	6	-0.000001265	-0.000000845	0.000000086
30	6	0.000000439	-0.000000251	-0.000000169
31	9	0.000000069	-0.000001154	0.000003556
32	1	-0.000002260	-0.000002301	0.000002371
33	1	-0.000000076	0.000000242	-0.000000433
34	1	0.000001516	0.000000227	-0.000000112
35	1	-0.000001005	0.000000587	0.000001804
36	1	0.000000220	0.000001306	0.000000578
37	1	-0.000000153	0.000000984	0.000001166
38	1	0.000000201	-0.000000060	-0.000001059
39	1	-0.000000290	0.000000249	-0.000000096
40	1	0.000000076	0.000000255	0.000000883
41	1	0.000000357	0.000000242	0.000000419
42	1	-0.000000088	-0.000000271	0.000000266
43	1	0.000000271	-0.000000059	0.000000427
44	1	0.000000242	-0.000000206	-0.000000274
45	1	-0.000000188	0.000000154	-0.000000033
46	1	-0.000000597	-0.000000174	0.000000303
47	1	-0.000000438	-0.000000047	0.000000246
48	1	0.000000051	0.000000100	0.000000155
49	1	0.000000045	-0.000000146	0.000000136
50	1	0.000000042	-0.000000114	0.000000313

Compound 19

$C_{27}H_{25}FN_4O_4$

Center Number	Atomic Number	X	Y	Z
1	6	-0.000000888	0.000001357	-0.000002013
2	6	-0.000002497	-0.000005505	0.000000315
3	6	0.000000246	0.000005990	-0.000001429
4	6	0.000001403	0.000001838	-0.000003550
5	6	0.000000650	0.000001583	0.000001277
6	6	-0.000000028	0.000001197	0.000000952
7	6	0.000002607	-0.000000641	0.000000798
8	6	-0.000003680	0.000000518	-0.000001425
9	6	-0.000003756	-0.000000593	-0.000003593
10	8	0.000002388	-0.000000885	0.000000634
11	8	-0.000005434	0.000002225	-0.000000988
12	8	-0.000001044	-0.000000867	0.000003385
13	7	0.000001181	0.000001826	-0.000002773
14	6	0.000001248	0.000000201	0.000001099
15	6	-0.000001257	0.000001378	-0.000000275
16	7	0.000001719	0.000006366	0.000001771

17	6	0.000003085	-0.000004868	-0.000002967
18	6	-0.000001672	-0.000000634	-0.000000842
19	6	-0.000003112	-0.000001105	-0.000001623
20	7	0.000000815	-0.000000681	0.000000655
21	6	-0.000002099	-0.000002668	0.000002484
22	6	0.000000075	0.000001079	0.000000498
23	6	-0.000000500	-0.000000013	0.000002179
24	6	-0.000000512	-0.000000072	0.000002732
25	6	-0.000000086	-0.000001140	0.000003131
26	6	0.000000200	-0.000001320	0.000004570
27	6	-0.000000239	-0.000001604	0.000003291
28	6	0.000000843	-0.000002445	0.000002827
29	6	0.000000590	-0.000000171	0.000001792
30	8	0.000000377	-0.000002060	0.000001856
31	9	-0.000001769	0.000003062	-0.000000057
32	1	0.000000866	0.000000846	-0.000002085
33	1	-0.000001466	0.000001694	0.000000412
34	1	0.000003182	0.000000443	0.000001316
35	1	-0.000001137	0.000002407	0.000000764
36	1	-0.000000691	0.000001341	-0.000001902
37	1	-0.000001463	0.000000445	0.000000568
38	1	-0.000000037	0.000000857	-0.000000098
39	1	0.000000398	0.000000030	-0.000001440
40	1	-0.000001043	0.000001358	-0.000002375
41	1	0.000000263	-0.000000671	-0.000001427
42	1	0.000000437	0.000001206	-0.000002423
43	1	0.000000312	-0.000000108	-0.000000498
44	1	0.000000820	0.000000604	-0.000000519
45	1	-0.000000368	0.000001168	0.000000538
46	1	-0.000000725	0.000000688	0.000002369
47	1	-0.000000500	-0.000000124	0.000003938
48	1	-0.000000095	-0.000001531	0.000004666
49	1	0.000000507	-0.000002495	0.000003914
50	1	0.000000544	-0.000001962	0.000001477
51	6	0.000000489	-0.000000788	-0.000000828
52	1	0.000000696	-0.000000258	-0.000003011
53	7	0.000001726	0.000001101	-0.000001206
54	6	0.000000473	-0.000000746	-0.000002934
55	1	0.000001408	-0.000001806	-0.000002024
56	6	0.000000415	-0.000001615	-0.000002710
57	1	0.000000952	-0.000001553	-0.000001571
58	1	0.000001030	-0.000001428	-0.000003361
59	1	0.000001216	-0.000001569	-0.000002702
60	6	0.000001312	0.000000160	0.000000214
61	1	0.000001628	0.000000956	-0.000001769

Compound 20

$C_{27}H_{25}FN_4O_5$

-

Center Number	Atomic Number	X	Y	Z
1	8	-0.000002738	-0.000004718	-0.000001759
2	6	0.000006165	-0.000000665	0.000003307
3	7	-0.000002445	0.000001800	0.000001022
4	6	0.000002786	0.000004268	-0.000004583
5	6	-0.000001489	-0.000005757	0.000001740
6	6	-0.000005657	0.000005745	-0.000004629
7	6	0.000000958	-0.000001736	0.000004493
8	6	0.000002970	0.000002002	-0.000005262
9	6	-0.000005713	-0.000014145	0.000009352
10	6	0.000001724	0.000008993	-0.000007055
11	6	0.000002050	-0.000002972	-0.000000427
12	6	-0.000002478	0.000003211	-0.000001150
13	6	0.000004590	-0.000004819	0.000005550
14	6	-0.000001804	0.000008173	-0.000000609
15	8	0.000000276	-0.000003210	-0.000000460

16	8	-0.000000196	-0.000005386	0.000002076
17	8	0.000001776	0.000010176	-0.000002408
18	6	-0.000002080	-0.000001193	-0.000000578
19	7	0.000000790	0.000006043	-0.000008864
20	6	0.000004760	0.000001220	0.000002863
21	6	-0.000004886	0.000001349	0.000001184
22	7	-0.000001663	-0.000005151	-0.000002958
23	6	0.000008625	0.000005220	-0.000000750
24	6	-0.000006141	-0.000002906	-0.000002260
25	6	0.000004146	-0.000001656	-0.000000349
26	7	0.000000091	-0.000003639	0.000004692
27	6	-0.000006036	0.000003634	-0.000005176
28	6	0.000001532	0.000003990	-0.000001144
29	6	0.000000666	-0.000004981	0.000001995
30	6	0.000002809	0.000001049	-0.000004189
31	6	-0.000003281	-0.000001214	0.000003938
32	6	-0.000001856	-0.000003212	-0.000003707
33	6	0.000002590	0.000002645	0.000002157
34	6	-0.000001951	-0.000001053	0.000008443
35	6	0.000001926	0.000002903	-0.000006930
36	8	0.000000085	-0.000000784	-0.000000850
37	9	-0.000000647	-0.000001155	0.000000572
38	1	0.000000239	-0.000000438	-0.000000204
39	1	-0.000001643	0.000000761	-0.000000206
40	1	0.000000791	-0.000001737	0.000003639
41	1	-0.000000738	0.000000027	0.000000118
42	1	-0.000001399	0.000000397	0.000001742
43	1	0.000003794	-0.000000946	-0.000000593
44	1	-0.000000166	0.000001054	0.000000590
45	1	0.000001122	-0.000000541	-0.000000843
46	1	-0.000000092	-0.000000514	0.000000129
47	1	0.000000319	0.000000825	-0.000000242
48	1	-0.000000238	-0.000001056	0.000000029
49	1	-0.000000219	0.000000159	-0.000000088
50	1	0.000001573	-0.000000007	0.000001223
51	1	-0.000001138	-0.000000325	-0.000001177
52	1	-0.000002111	0.000000113	0.000002909
53	1	0.000000417	0.000000832	0.000003364
54	1	0.000001576	-0.000001271	0.000001008
55	1	0.000000157	-0.000000188	0.000000593
56	1	-0.000002454	-0.000000462	0.000000156
57	1	-0.000000183	-0.000000062	0.000001791
58	1	-0.000000494	0.000000719	-0.000000222
59	1	-0.000000215	0.000000461	-0.000000330
60	1	0.000000323	-0.000000205	-0.000000048
61	1	0.000000183	-0.000000027	0.000000003
62	1	0.000000341	0.000000362	-0.000000627

-
